# Microbial Dysbiosis Linked to Metabolic Dysfunction-Associated Fatty Liver Disease in Asians: *Prevotella copri* Promotes Lipopolysaccharide Biosynthesis and Network Instability in the Prevotella Enterotype

**DOI:** 10.3390/ijms25042183

**Published:** 2024-02-11

**Authors:** Heng Yuan, Xuangao Wu, Xichun Wang, Jun-Yu Zhou, Sunmin Park

**Affiliations:** 1 Department of Bioconvergence, Hoseo University, Asan 31499, Republic of Korea; yuanheng.changan@gmail.com (H.Y.); niyani0@naver.com (X.W.); zjy888zjy888@gmail.com (J.-Y.Z.); 2 Department of Computer and Data Analysis, Northern Arizona University, Flagstaff, AZ 86011, USA; steve.wang@nau.edu; 3 Department of Food and Nutrition, Obesity/Diabetes Research Center, Hoseo University, Asan 31499, Republic of Korea

**Keywords:** Asian MAFLD, gut microbiota, enterotype, microbial biomarkers

## Abstract

Metabolic dysfunction-associated fatty liver disease (MAFLD), formerly known as non-alcoholic fatty liver disease (NAFLD), is characterized by hepatic fat accumulation by metabolic dysfunction. The rising prevalence of MAFLD, especially among Asians, may be associated with changes in gut microbiota. We investigated gut microbiota characteristics and potential mechanisms leading to MAFLD development according to enterotypes. Case-control studies examining the gut microbiota composition between MAFLD and non-MAFLD participants were searched in public databases until July 2023. Gut microbiota was categorized into two enterotypes by principal component analysis. According to the enterotypes, LEfSe, ALDEx2, XGBoost, and DCiPatho were utilized to identify differential abundances and pathogenic microbes in the gut between the MAFLD and non-MAFLD groups. We analyzed microbial community networks with the SprCC module and predicted microbial functions. In the Prevotella enterotype (ET-P), 98.6% of Asians and 65.1% of Caucasians were associated with MAFLD (*p* = 0.049). MAFLD incidence was correlated with enterotype, age, obesity, and ethnicity (*p* < 0.05). Asian MAFLD patients exhibited decreased Firmicutes and *Akkermansia muciniphila* and increased Bacteroidetes and *P. copri*. The pathogenicity scores were 0.006 for *A. muciniphila* and 0.868 for *P. copri*. The Asian MAFLD group showed decreased stability and complexity in the gut microbiota network. Metagenome function analysis revealed higher fructose metabolism and lipopolysaccharide (LPS) biosynthesis and lower animal proteins and α-linolenic acid metabolism in Asians with MAFLD compared with the non-MAFLD group. LPS biosynthesis was positively correlated with *P. copri* (*p* < 0.05). In conclusion, *P. copri* emerged as a potential microbial biomarker for MAFLD. These findings enhance our understanding of the pathological mechanisms of MAFLD mediated through the gut microbiota, providing insights for future interventions.

## 1. Introduction

Non-alcoholic fatty liver disease (NAFLD) is a disease characterized by abnormal fat accumulation in the liver without extensive alcohol intake (>20 g/day), and it has recently been revised into metabolic dysfunction-associated fatty liver disease (MAFLD) [1]. The new terminology emphasizes the link between hepatic fat accumulation and metabolic disturbances, such as obesity, insulin resistance, and metabolic syndrome, beyond the association with alcohol consumption [1]. While there is a global increase in the prevalence of MAFLD, this increase is particularly notable in the Asian region [2]. The substantial rise in MAFLD patients in countries like South Korea and China within the Asian regions is likely linked to rapidly changing lifestyles and suboptimal dietary practices, potentially involved in gut microbiota changes [2,3]. It is further related to weight changes. However, Asians have a lower cutoff of obesity to induce metabolic syndrome and MAFLD than Caucasians, potentially related to their specific differences. Therefore, studying the differences between different ethnic groups and their impact on the development of MAFLD is crucial for a deeper understanding of the mechanisms of MAFLD and for devising more effective prevention and treatment strategies.

With the advancement of sequencing technologies like next-generation sequencing (NGS), there is growing evidence of a close association between MAFLD and the gut microbiota [4,5]. The gut microbes can be classified into three enterotypes based on their compositions and characteristics: Prevotella (ET-P), Bacteroides (ET-B), and Ruminococcus (ET-R) [6]. Asians have a relatively higher proportion of ET-P than Caucasians. Interestingly, although individuals do not strictly fall into ET-P, they have approximately 10–20% *Prevotella copri* (*P. copri*) of gut microbiota, possibly linked to specific dietary practices [7,8]. *P. copri* plays a distinctive role in carbohydrate metabolism and is known for efficient carbohydrate digestion, including sugar and fiber, and the high production of propionic acid, one of the short-chain fatty acids (SCFAs) [9]. Moreover, individuals in ET-P tend to have a lower energy intake than their estimated energy requirement but have a higher proportion of metabolic syndrome [6]. Nevertheless, Francisco et al. propose a plausible association between an abundance of *P. copri* and an elevated risk of insulin resistance [10]. Additionally, *Prevotella* can generate higher levels of endotoxin lipopolysaccharide (LPS), a component of bacterial outer membranes [11]. Elevated LPS levels may enter the bloodstream, triggering inflammation and fostering the development of MAFLD [12]. Additionally, the interaction between environmental factors, gut microbiota, and the intestinal epithelium is essential for maintaining the integrity of the gut barrier. The function of this barrier is to prevent the penetration of harmful substances and pathogens, thus protecting the body from infections and inflammation [13]. LPS may enter the bloodstream when the gut barrier is compromised, triggering immune responses and inflammation. Prolonged inflammatory responses can lead to various health issues, including insulin resistance and MAFLD [13]. 

Examining the roles of opportunistic gut microbiota in MAFLD development is crucial, specifically focusing on contrasting gut microbiota compositions and MAFLD mechanisms across different enterotypes. This study aims to pinpoint gut microbial markers linked to MAFLD and enhance the comprehension of MAFLD pathophysiology based on enterotypes. The goal is to offer more precise treatment and preventive strategies tailored to enterotypes, particularly on ET-P.

## 2. Results

### 2.1. Screening and Studies Included in the Analysis 

**Figure 1:**
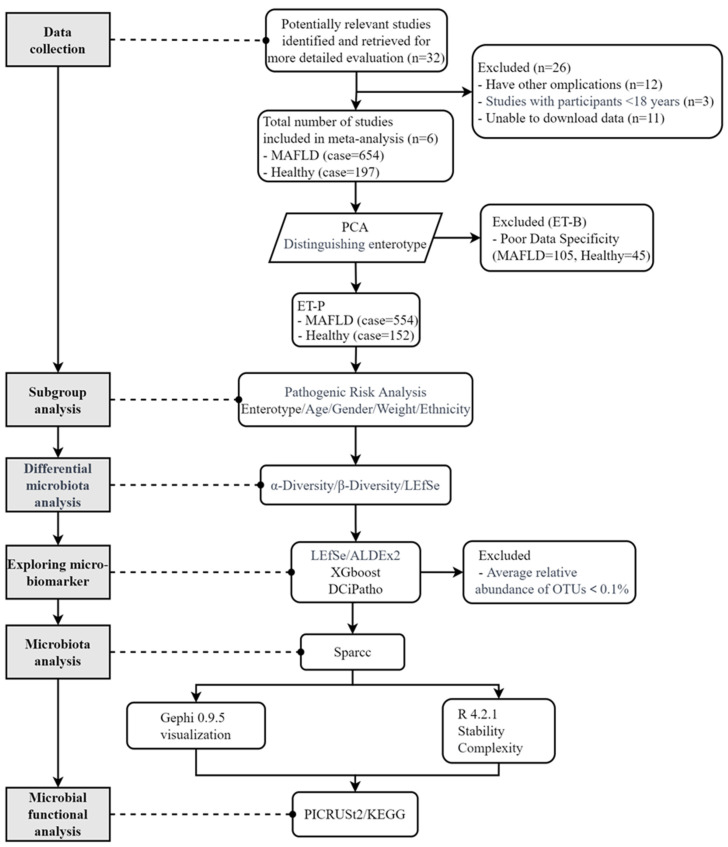
Flowchart of data selection and analysis in this study. PCA: principal component analysis; ET-B: enterotype—Bacteroides; ET-P: enterotype—Prevotella; MAFLD: metabolic dysfunction-associated fatty liver disease; LEfSe: linear discriminant analysis effect size; ALDEx2: ANOVA-Like Differential Expression tool; XGBoost: eXtreme Gradient Boosting; DCiPatho: deep cross-fusion networks for identification of pathogens; OTU: operational taxonomic unit; SparCC: sparse correlations for compositional data; PICRUSt2: Phylogenetic Investigation of Communities by Reconstruction of Unobserved States 2; KEGG: Kyoto Encyclopedia of Genes and Genomes.

The screening process is delineated in Figure 1. Each study met the criteria for MAFLD based on the new fatty liver definition [1]. In each study, MAFLD was diagnosed with imaging or the biopsy technique. Additionally, if the study had sufficient exclusion criteria, it was included. From an initial search, 32 studies were identified, with 26 excluded for various reasons. The excluded studies comprised 12 studies with other disease complications, 3 involving participants <18 years old, and 11 that could not be included due to the non-availability of gut microbiota data. Eventually, six studies were incorporated into the analysis. The essential characteristics of the included studies are summarized in Table 1. According to the optimal number of clusters (Supplementary Figure S1A), the participants were classified into two enterotypes (Supplementary Figure S1B). ET-1 and ET-2 primarily contained Bacteroidaceae and Prevotellaceae at the family level (Supplementary Figure S2A). They mainly included *Bacteroides* and *Prevotella* in the genus level (Supplementary Figure S2B). ET-1 corresponds to ET-B, and ET-2 represents ET-P.

A thorough evaluation of data quality for the two enterotypes, specifically about the MAFLD cases and non-MAFLD (Healthy) controls, was executed using XGBoost, and the assessment outcomes are elucidated in Supplementary Table S1. Notably, the specificity of ET-B was determined to be 0.748, signifying that the data for ET-B lacked a clear distinction between positive and negative classes. Furthermore, due to insufficient data for training a deep learning model, ET-B was ultimately excluded from this study (Supplementary Figure S3) [14]. The final analysis comprised 706 gut microbiota datasets, containing 554 cases of MAFLD and 152 non-MAFLD controls. The population distribution included 282 Asians and 424 Caucasians. Detailed clinical characteristics are provided in Supplementary Table S2.

### 2.2. Multifactorial Analysis Revealed Elevated MAFLD Risk in ET-P and Ethnicity-Associated Microbiota Dynamics

**Figure 2:**
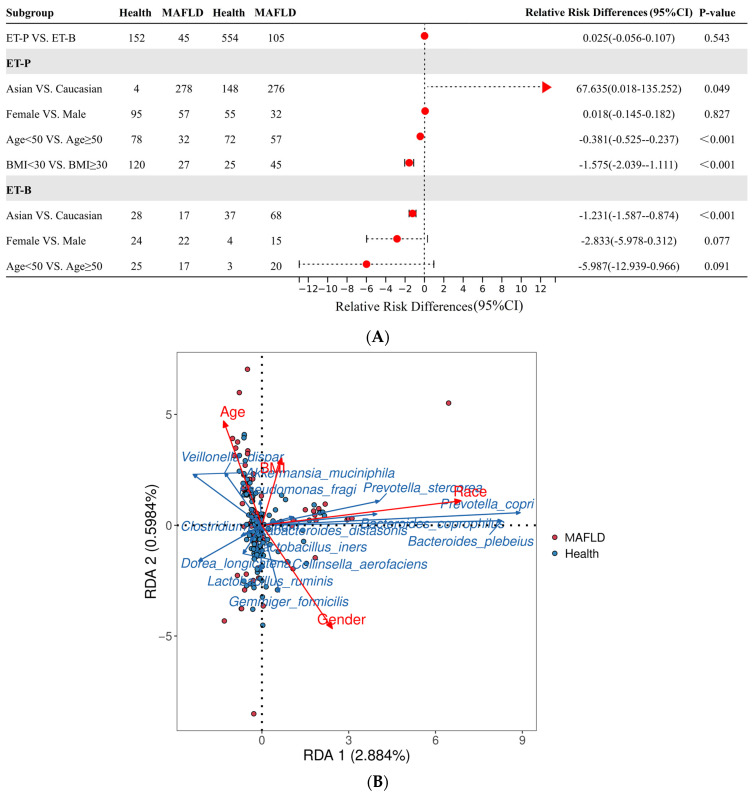
Risk assessment of multifactorial clinical features in metabolic dysfunction-associated fatty liver disease (MAFLD) and its relationship at the microbial species level. (**A**). Forest plot of multifactorial clinical feature risk assessment. (**B**). The mapping relationship between clinical features and intestinal microbiota at the species level by redundancy analysis (RDA). Red arrows represent clinical features, blue arrows represent microorganisms, red dots denote the data of the MAFLD group, and blue dots denote the data of the non-MAFLD group. MAFLD: metabolic dysfunction-associated fatty liver disease; CI: confidence interval; ET-B: enterotype—Bacteroides; ET-P: enterotype—Prevotella; PCA: principal component analysis.

Figure 2A depicts the evaluation of multifactorial pathogenic risks. It indicates no statistical difference in the MAFLD incidence between the ET-P and the ET-B groups (*p* = 0.543). The subgroup analysis further revealed significant risk factors for the development of MAFLD, including Asian ethnicity, obesity, and advanced age (*p* < 0.05), while gender appeared to be unrelated (*p* = 0.827) in ET-P. Particularly noteworthy is the observation that within the ET-P group, individuals of Asian ethnicity exhibited an incidence rate 67.635 times higher than that of Caucasians (*p* = 0.049); due to the small number of healthy Asians in the ET-P group, a Bayesian model sensitive to small sample sizes was used for posterior analysis. The results showed that the estimated value for the target metric in the Asian population (mean of 0.98) was much higher than that in the Caucasian population (mean of 0.65). The model indicated a notable distinction in gut microbiota between Asians and Caucasians having ET-P, as demonstrated by the narrow 95% confidence intervals and strong convergence (Rhat = 1) (Supplementary Figure S3). The results suggested that Asians with ET-P were a high-risk population. Conversely, in the ET-B group, MAFLD risk in Asians was negatively associated 1.231 times with that in Caucasians (*p* < 0.001).

The relationship between various MAFLD risks and ET-P microbiota was mapped with redundancy analysis (RDA). In this depiction, on the PCA plot, red arrows signify risk factors, blue arrows denote the primary microbiota community, and red and blue dots represent the mapping of the MAFLD and non-MAFLD groups, respectively (Figure 2B). An angle less than 90 degrees suggests a positive correlation, with the length indicating the strength of the influence. The results revealed a positive correlation between age and BMI (<90°), irrespective of ethnicity (≈90°). Importantly, ethnicity exerts the most substantial influence on disease, with the *Prevotella* genus, mainly including *P*. *copri* and *P*. *stercorea*, and the *Bacteroides* genus, including *Bacteroides plebeius* and *Bacteroides coprophilus*, representing microbiota that was positively correlated with race, wherein *P*. *copri* was the most significant differentiating species between the ethnicities. Furthermore, increased BMI and age were associated with an abundance of *Pseudomonas fragi*, while the abundance of *Veillonella dispar* was correlated positively with age (Figure 2B).

### 2.3. Changes in Microbial Community Composition in MAFLD based on Alpha Diversity, Beta Diversity, and Taxonomic Analysis

**Figure 3:**
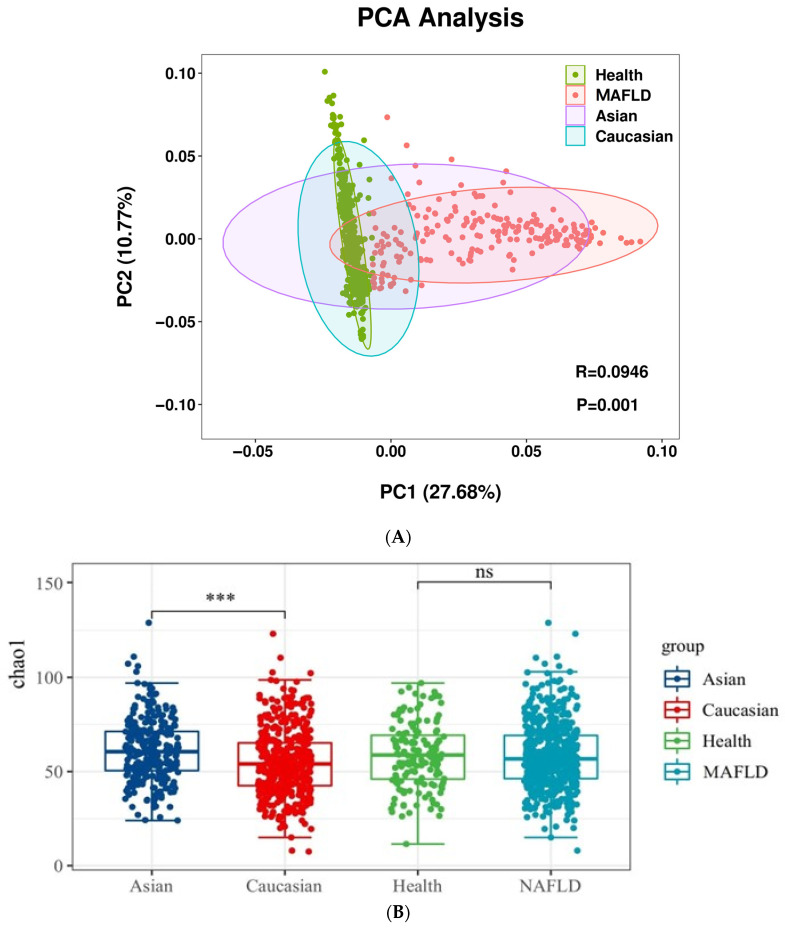

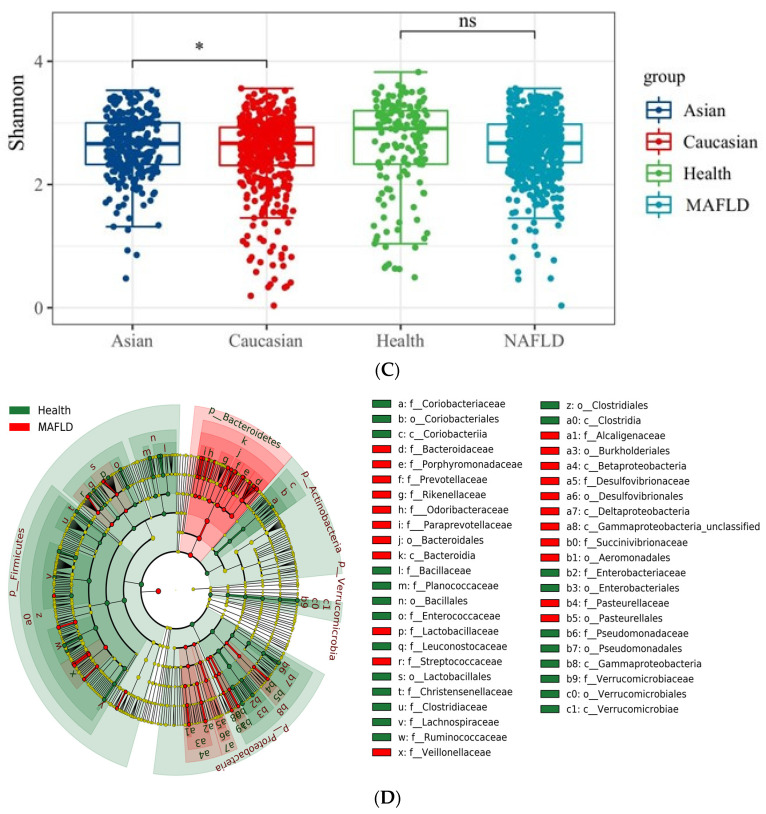
Analysis of changes in the gut microbiota of metabolic dysfunction-associated fatty liver disease (MAFLD) (**A**). Highly abundant gut microbiota in the transition between the non-MAFLD state and MAFLD, as well as between Asian and Caucasian populations, visualized through reduced dimensionality in redundancy analysis (RDA). (**B**). Differences in the Chao1 index depicting alpha diversity between the ethnic groups. (**C**). Differences in the Shannon index depicting alpha diversity between the ethnic groups. (**D**). Evolutionary branching diagram depicting changes in the taxonomic hierarchy from phylum to family for gut microbiota in MAFLD patients. Red indicates a higher relative abundance in the MAFLD group, while green represents the Healthy group (only showing linear discriminant analysis (LDA) scores (Log 10) > 2). * *p*-value < 0.05, *** *p*-value < 0.001, ns: not significant.

In the PCA analysis, beta diversity revealed significant differences in the main composition of the gut microbiota between the MAFLD and non-MAFLD groups. The confidence ellipse for Asians encompassed more MAFLD than that for Caucasians, suggesting that bacteria with a high abundance in Asian MAFLD patients were primarily concentrated in those with a high abundance in MAFLD. In contrast, the opposite is true for Caucasians (Figure 3A). Specifically, PC1 and PC2 explained 27.68% and 10.77% of the gut microbiota variability, totaling 38.45%. Although the model showed that the specific variables and between-group differences were statistically significant (*p* = 0.001), the correlation was weak (R = 0.0946), indicating that there might be more complex structures in the gut microbiota to be further elucidated. The Chao and Shannon indexes, representing the α diversity of the gut microbiota, were not significantly different between the MAFLD and non-MAFLD groups (Figure 3B,C). 

Figure 3D depicts the evolutionary branching of the gut microbiota in MAFLD patients, spanning taxonomic classifications from phylum to family. The red segments indicate a higher relative abundance of microbiota in the MAFLD group than the Healthy group (LDA score (Log 10) > 2), while the green segments represent a lower relative abundance. MAFLD patients exhibited an increase in Bacteroidetes and a decrease in Firmicutes. Proteobacteria displayed inconsistent changes: Beta-proteobacteria, Delta-proteobacteria, and Gamma-proteobacteria, including Pasteurellales and Aeromonadales, increased, while Pseudomonadales and Enterobacteriales decreased in patients with MAFLD. Within Verrucomicrobia, only Verrucomicrobiaceae decreased in patients with MAFLD. As outlined in Table 2, *Prevotella*, *Parabacteroides*, *Dialister*, and *Bacteroides* showed a higher relative abundance in MAFLD compared with the Healthy group, while *Blautia* exhibited the opposite trend. 

### 2.4. Identification of Potential Microbiota Biomarkers

**Figure 4:**
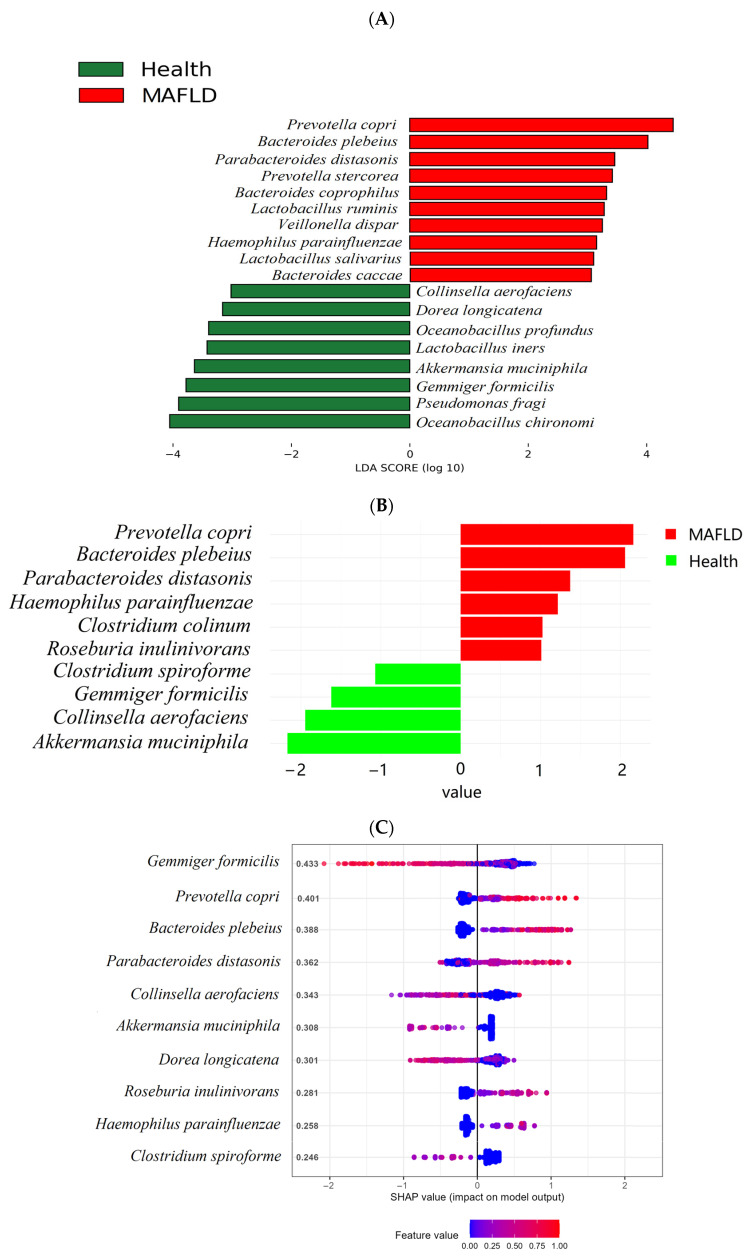

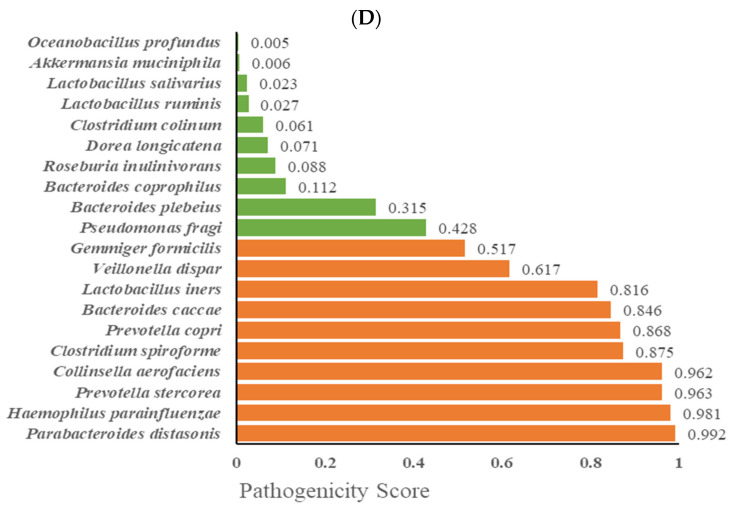
Process of selecting core pathogenic microbial markers. (**A**). LEfSe conducts differential abundance analysis. (**B**). ALDEx2 complements the analysis of differentially abundant microbes. (**C**). Xgboost characterizes these differentially abundant microbes by feature values (ROC AUC: 0.9890937). (**D**). DCiPatho evaluates the toxicity of these differentially abundant microbes. Abbreviations: LEfSe: linear discriminant analysis effect size; ALDEx2: ANOVA-Like Differential Expression tool for high throughput sequencing data; Xgboost: eXtreme Gradient Boosting; ROC AUC: receiver operating characteristic area under the curve; DCiPatho: deep cross-fusion networks for genome-scale identification of pathogens.

As shown in Figure 4, higher LDA scores were observed for *P. copri*, *Bacteroides plebeius, Parabacteroides distasonis, P. stercorea, Bacteroides coprophilus, Lactobacillus ruminis, Veillonella dispar, Haemophilus parainfluenzae, Lactobacillus salivarius*, and *Bacteroides caccae* using LEfSe (Figure 4A). Additionally, elevated ALDEx scores were noted for *P. copri*, *Bacteroides plebeius, Parabacteroides distasonis, Haemophilus parainfluenzae, Clostridium column, Roseburia inulinivorans*, and *P. stercorea* in the MAFLD group (Figure 4B). 

Subsequently, the XGBoost SHAP analysis of the 12 bacteria identified by LEfSe and ALDEx was conducted, and Figure 4C includes the top 10 SHAP values. The XGBoost model demonstrated an outstanding performance with a specificity of 0.992, a sensitivity of 0.990, and an ROC AUC of 0.95 (Supplementary Figure S4), affirming its reliability in predictive tasks. Specifically, the analysis revealed that *P. copri, B. plebeius, P. distasonis, R. inulinivorans*, and *H. parainfluenzae* were visually represented as red dots on the right side of the zero-line concerning MAFLD. This indicated a positive correlation with the MAFLD state. In contrast, *G. formicilis, C. aerofaciens, A. muciniphila, D. longicatena,* and *C. spiroforme* exhibited an opposite pattern. These findings suggest a discernible association between the selected bacteria and MAFLD. 

Concurrently, DCiPatho, which incorporates the frequency features of three to seven k-mers through deep cross-fusion networks for pathogen detection, was used, making it instrumental in screening critical pathogenic microbes. As observed in Figure 4D, *Bacteroides plebeius* displayed a pathogenicity score of 0.315, which is typically considered safe as the score is below 0.5. In contrast, *P. copri* was identified as a microbe with higher toxicity, exhibiting a score of 0.868. Consequently, *P. copri* was recognized as a potential pathogenic microbial biomarker for MAFLD.

### 2.5. The Intestinal Microbiota Relationship Network and the Differences in P. copri in the Network Based on Ethnicities

**Figure 5:**
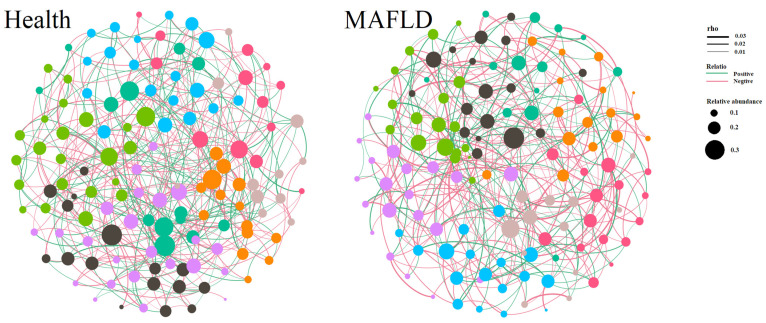
Correlation analysis and functional prediction of intestinal microbiota in metabolic dysfunction-associated fatty liver disease (MAFLD). Network diagrams of intestinal microbiota between the non-MAFLD and MAFLD groups are demonstrated. The color of nodes represents communities automatically divided by Gephi software based on correlations; the width of the connecting lines represents the magnitude of the correlation; green lines represent positive correlations; red lines represent negative correlations; and the size of the nodes represents the average relative abundance of the respective microorganisms.

The correlation analysis of gut microbiota in non-MAFLD individuals and patients with MAFLD using SparCC led to the network analysis (Figure 5) and the derivation of various metrics describing the network properties (Supplementary Table S4). In comparison with the non-MAFLD group, the MAFLD group exhibited a sparser microbial network (MAFLD 432 vs. Healthy 404) with lower average node connectivity (MAFLD 5.82 vs. Healthy 6.60), suggesting more dispersed and weakened interactions among microorganisms. The longer average path length indicated a slower substance exchange within the network (MAFLD 1.17 vs. Healthy 0.79). Additionally, after removing 50% of the nodes, the MAFLD network displayed lower robustness (MAFLD: 0.42 vs. non-MAFLD: 0.47), indicating the relative fragility of the network. 

The interaction analysis focused on the relationship between *P. copri* and other gut bacteria within Asian and Caucasian populations, as depicted in Supplementary Figure S6 and detailed in Supplementary Table S4. Within the ET-P group, *P. copri* displayed a significantly higher weighted degree in the intestinal microbiota of Asians compared with Caucasians (Asians: 14.13 vs. Caucasians: 6.59) despite a similar relative abundance of *P. copri* across ethnicities (Asians: 36.36% vs. Caucasians: 31.82%). *P. copri* in Asians with ET-P acted as a central bacterium, fostering more interactions with other microbes. Additionally, the clustering coefficient of *P. copri* was higher in Asians (Asians: 0.41 vs. Caucasians: 0.26), and its PageRank value in Asians (0.0153) surpassed that in Caucasians (0.0065), emphasizing its central role within the intestinal microbiota network in Asian populations. These findings suggest that in Asian ET-P, *P. copri* may hinder the proliferation of certain beneficial bacteria, such as *Bifidobacterium*, potentially contributing to gut dysbiosis.

### 2.6. Metagenome Function of Gut Microbiota 

**Figure 6:**
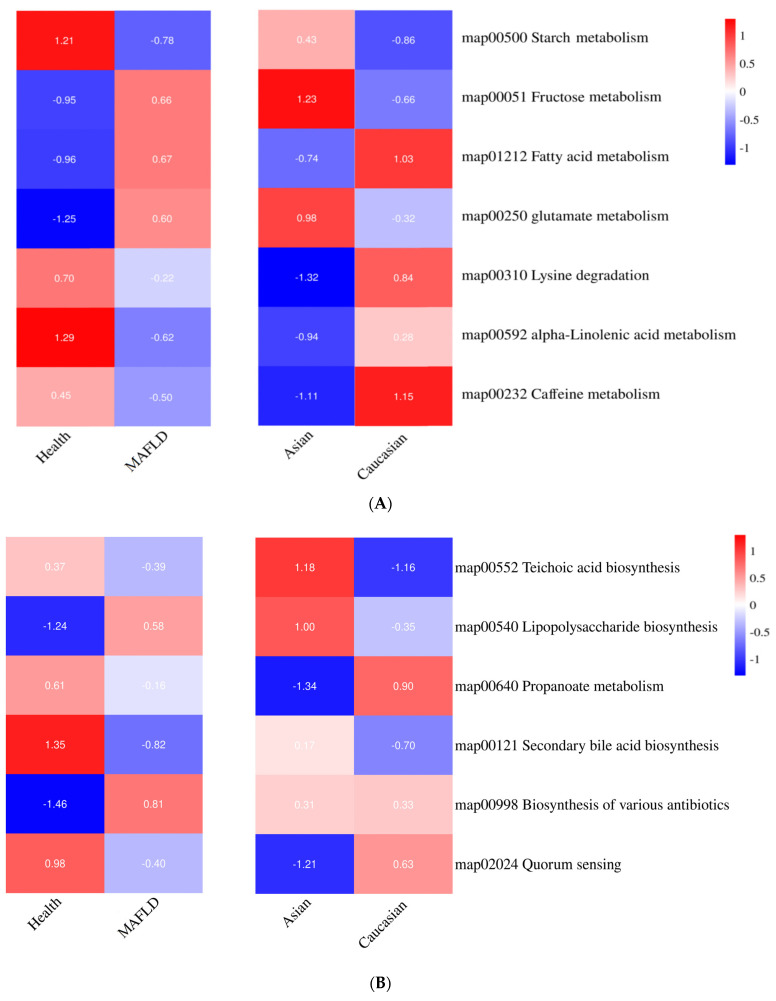
Comparative analysis of gut microbiota functions between Healthy individuals and metabolic dysfunction-associated fatty liver disease (MAFLD) patients in Asian and Caucasian populations. (**A**). Differences in nutritional metabolism (**B**). Variations in metabolic by-product data. The data were normalized by Z-score.

Functional predictions of the gut microbiota were conducted using PICRUSt2. Figure 6A illustrates the characteristics of the nutrient metabolism of the gut microbiota in Asians compared to those in Caucasians and non-MAFLD individuals relative to those with MAFLD. Regarding carbohydrate metabolism, the gut microbiota in Asians exhibited higher starch (KEGG ID: 00500) and fructose (KEGG ID: 00051) metabolism compared with Caucasians. Furthermore, fructose metabolism was positively correlated with MAFLD (MAFLD 0.66 vs. non-MAFLD −0.95). Concerning lipid metabolism, the gut microbiota in Caucasians exhibited higher fatty acid metabolism (KEGG ID: 01212) compared with Asians. Lipid metabolism was positively correlated with MAFLD (MAFLD: 0.67 vs. non-MAFLD: −0.96). In protein metabolism, the Asian gut microbiota exhibited a higher metabolism of glutamate (KEGG ID: 00250) than the Caucasian gut microbiota, while the Caucasian microbiota predominantly metabolized lysine (KEGG ID: 00310). Among the other nutrients, the Asian gut microbiota demonstrated lower alpha-linolenic acid (KEGG ID: 00592) and caffeine (KEGG ID: 00232) metabolism compared with the Caucasian gut microbiota. The metabolic features of the gut microbial community are depicted in Figure 6B, and elevated levels of exogenous teichoic acid and LPS in Asians (KEGG ID: 00540) can be observed. The MAFLD group showed no correlation with teichoic acid (KEGG ID: 00552). However, propionate metabolism was higher in the Healthy group than in the MAFLD (KEGG ID: 00640) group, while acetate and butyrate metabolism and biosynthesis exhibited no significant differences between the MAFLD and Healthy groups. The Healthy group displayed increased levels of secondary bile acids, whereas Caucasians showed a lower synthesis of secondary bile acids (KEGG ID: 00121). The biosynthesis of various antibiotics, potentially leading to microbial imbalance contributing to MAFLD, exhibited no significant difference between Asians and Caucasians (KEGG ID: 00998). Finally, quorum sensing, a process of microbial communication via signaling molecules crucial for maintaining the structure and function of microbial communities, appeared reduced in MAFLD, indicating weakened communication and interaction within the microbial community. Moreover, the lower quorum sensing values in Asians may be related to the incidence of MAFLD (KEGG ID: 02024).

**Figure 7:**
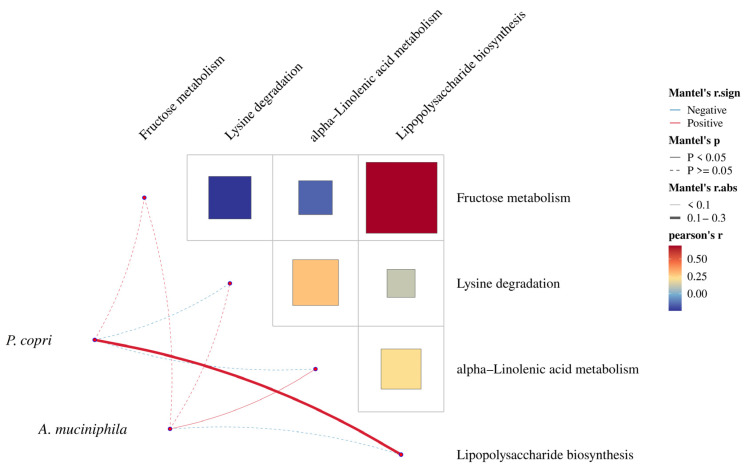
Differential analysis of microbial synthesis metabolites in the gut microbiota between the two groups.

The analysis of the association between the abundance of *P. copri* and *Akkermen cia muciniphila* with the metabolism of fructose, leucine, alpha-linolenic acid, and LPS, which are highly relevant to MAFLD, revealed a positive correlation between *P. copri* abundance and fructose metabolism and LPS biosynthesis. However, *Akkermencia muciniphila* showed a positive correlation with alpha-linolenic acid metabolism (Figure 7).

## 3. Discussion

The present study indicated that ET-P, dominated by the genus *Prevotella*, constitutes a high-risk group for MAFLD in the Asian population. In contrast, ET-B shows a higher incidence rate among Caucasians. RDA further revealed that age and BMI were positively correlated with the risk of MAFLD. The racial factor significantly impacted the disease, although *P. copri* is a common gut microbiota in Asians and Caucasians [8]. The ET-P network in Asians might negatively affect MAFLD risk. The microbiota network analysis indicated that compared with Caucasians, *P. copri* occupied a more central position in the intestinal microbiota of Asians, which is a critical factor in its pathogenicity in the Asian population, with *P. copri* exhibiting an influential association with MAFLD in the Asian population. Therefore, the results suggested that the interaction between *P. copri* and other microbiota could serve as a potential biomarker for predicting and diagnosing MAFLD in Asians. 

*P. copri* plays a significant role in the initiation and progression of type 2 diabetes, mainly linked to insulin resistance and metabolic disturbances [15]. However, some research has demonstrated that *P. copri is associated with* substantial glucose and insulin tolerance improvement, especially in diets rich in fiber [16]. The impact of *P. copri* may be diet- and amount-dependent [17]. *P. copri* consumes carbohydrates, including sugar and dietary fibers, and sugars may be a more accessible source for *P. copri* than dietary fiber. Asians consuming a diet rich in white rice exhibit elevated levels of *P. copri*, which is positively linked to metabolic syndrome risk, potentially contributing to MAFLD [18]. This indicates that there are conflicting reports regarding the advantages of *P. copri*, making it an essential yet enigmatic member of the gut microbiome [19]. The contradictory results may be linked to *P. copri* abundance, carbohydrate source, dietary components, lifestyle, and the host’s genetic preposition. Type 2 diabetes and insulin resistance are closely related to MAFLD [20]. Various reports highlight its association with MAFLD [21,22], indicating that this microorganism may play a role in developing MAFLD through diverse pathways. 

Additionally, *P. copri* has been implicated in immune overactivation [23,24], possibly associated with MAFLD and its progression into hepatic steatosis. Increased levels of *P. copri* have been observed in individuals with rheumatoid arthritis [25] and inflammatory bowel diseases (IBD) [24]. This increase in *P. copri* can disrupt the normal function of the intestinal barrier and trigger abnormal activation of the gut’s immune response. Such changes not only aggravate the symptoms associated with IBD but may also heighten the risk of developing colorectal cancer [26]. Consequently, variations in *P. copri* levels are significant not merely as indicators of IBD but also in their potential contribution to the onset and progression of colorectal cancer, underlining their critical role in both conditions [26]. Studies exploring the overall microbial variations in patients with MAFLD reveal an increase in *P. copri* [27], although the reasons for these findings remain unclear. One possible explanation is that dietary habits, inflammatory status, and age may confound the relationship between *P. copri* and susceptibility to MAFLD [28]. Another potential explanation could be the differential effects of *P. copri* under various genetic backgrounds [29]. A review suggests that, in Asians, MAFLD is commonly found in non-obese individuals, referred to as “lean” MAFLD [30]. Despite having normal weight, these patients exhibit patterns of insulin resistance and distribution of free fatty acids similar to those in obese individuals, which may be related to gut microbiota [31]. The results of this study indicate a positive correlation with LPS biosynthesis in MAFLD patients, suggesting that the microbiota might exacerbate the development of MAFLD through increased immune activation and inflammation. 

The present study did not include liver steatosis or fibrosis, and the impact of *P. copri* was mainly involved in MAFLD. However, a recent study reported that *P. copri* significantly increases in the advanced stages of liver fibrosis in chronic liver disease, particularly in MAFLD patients, suggesting its potential as a non-invasive biomarker for MAFLD [32]. The association between *P. copri* and the progression of liver fibrosis offers new directions for future therapeutic interventions for MAFLD. It supports the potential role of gut microbiota in liver fibrosis [32]. The function of *P. copri* is complex and influenced by various factors such as dietary habits, inflammatory status, age, and genetic background. Future research needs to delve deeper into its role in the gut microbiome and its overall impact on health. 

Furthermore, further research is necessary to comprehensively understand the detailed pathogenic mechanisms of *P. copri* in MAFLD. In Caucasian populations, characterized by dietary habits rich in fats and animal proteins, the prevalence of *P. copri* might be lower [8]. This suggests a limited impact of *P. copri* abundance on MAFLD in Caucasians. *P. copri* is a significant component of the human microbiome, often associated with diets rich in plants [33]. While offering benefits such as improved glucose metabolism, they are also associated with disease conditions, notably opportunistic pathogens [34]. Moreover, studies have indicated that *P. copri* can disrupt the gut microbiome, decreasing beneficial fatty acids and increasing intestinal inflammation, thus highlighting its potential role in exacerbating autoimmune diseases [35]. 

The complexity and stability of the gut microbial network are crucial factors for maintaining eubiosis and preventing metabolic diseases, including MAFLD [36]. This study observed a simultaneous decrease in the complexity of the gut microbiota network and the inter-microbial correlations characterizing MAFLD patients. Reduced connectivity among microbes typically signifies weakened interactions within the microbial community, reflecting an unstable and imbalanced state. Such a state may impact adaptability to the external environment and the host’s health [37,38]. However, divergent views exist: simplifying the microbial network does not necessarily represent an unhealthy state but might be an adaptive change, especially in the early stages of certain chronic diseases [39]. Therefore, the relationship between the complexity of the microbial community network and the onset and development of MAFLD requires further investigation.

Functional predictions of the gut microbiota using PICRUSt2 revealed that the Asian gut microbiota exhibited increased activity in fructose metabolism, while the Caucasian gut microbiota showed enhanced lipid metabolism. These metabolic traits were concurrently found to be positively correlated with MAFLD. Excessive fructose consumption in the Asian diet, particularly with the widespread consumption of processed food [40], has significantly increased fructose intake [41]. Due to the limited capacity of the small intestine to absorb fructose [42], high doses easily reach the colon and induce bacterial fermentation [43]. In contrast to the metabolic pathway using ketohexokinase, gut microbiota phosphorylate fructose via hexokinase, initiating glycolysis [44]. Research by Todoric et al. demonstrated that long-term high-dose fructose intake increases intestinal permeability and endotoxemia in mice [45], triggering hepatic inflammatory responses and downstream cytokine pathways, thereby promoting fat synthesis and the development of fatty liver [46]. These findings reinforce the link between gut microbiota-mediated fructose metabolism, hepatic inflammation, and fatty liver [47]. Therefore, this study underscores the importance of reducing fructose intake among Asian MAFLD patients to mitigate the risk of MAFLD induced by active fructose metabolism.

Furthermore, this study clearly demonstrated that the overproduction of LPS by gut microbiota is a significant microbial aspect contributing to the susceptibility of Asians to MAFLD. Excessive production or accumulation of LPS in the gut damages this mucosal barrier, which is a protective barrier designed to prevent harmful bacteria and substances from entering the circulatory system [48]. LPS can activate immune cells such as macrophages and dendritic cells in the intestinal mucosa. This activation triggers an inflammatory response characterized by the release of pro-inflammatory cytokines, such as TNF-α and IL-1β, exacerbating inflammation and injury to the intestinal mucosa [48]. Moreover, LPS can alter the permeability of the intestinal mucosa, facilitating the ingress of bacteria and harmful substances into the bloodstream, thereby inducing a systemic inflammatory response [49]. Upon entering the bloodstream, LPS interacts with receptors in the immune system, including TLR-4, leading to an immune-mediated inflammatory response. This is particularly evident in the liver, where the activation of hepatic Kupffer cells results in liver inflammation [50]. Additionally, LPS may disrupt the insulin signaling pathway, leading to insulin resistance at the cellular level, thereby elevating blood glucose levels and contributing to the progression of MAFLD [51]. Therefore, the presence of LPS and its associated effects, including inflammation, intestinal mucosal damage, and impact on the insulin signaling pathway, collectively contribute to the progression of MAFLD.

Conversely, the risk of MAFLD in Caucasians is associated with decreased secondary bile acids. Studies suggest that reducing secondary bile acids may participate in the pathogenesis of MAFLD by affecting lipid metabolism, the balance of gut microbiota, and regulating metabolic pathways [52]. Secondary bile acids are transformed from primary bile acids by gut microbiota and play a crucial role in regulating gut microbiota composition and maintaining intestinal barrier integrity [53]. A decrease in secondary bile acids could lead to an imbalance in the gut microbiota, which in turn affects the integrity of the intestinal barrier, increases intestinal permeability, and thereby facilitates the entry of endotoxins, such as LPS, into the bloodstream, causing systemic inflammatory responses [53]. 

This study has some limitations. First, insufficient data hindered the analysis of the characteristics of Asian MAFLD in the ET-B group as data from 11 studies could not be downloaded, and the authors could not be contacted. This may lead to potential publication bias. Second, the data used in this study were not continuous follow-up data, making it challenging to elucidate the causal relationship between gut microbiota changes and the occurrence and development of MAFLD. Third, the number of healthy Asians with ET-P was only four. However, the data underwent Bayesian analysis and demonstrated good convergence, suggesting that the sample size was sufficient, but it still might include false positives. Finally, this study did not include a dietary survey. Therefore, the dietary recommendations based on gut microbiota functions might be biased. Despite these limitations, we are confident that the characterization of the pathological state of MAFLD patients using the features of these gut microbiota is accurate.

In summary, this study offers comprehensive insights into gut microbiota characteristics in the Asian population with MAFLD. The notable prevalence of ET-P among Asian MAFLD patients is intricately connected to traditional dietary patterns and features of the microbial community. Gut microbiota, particularly *P. copri*, appears to play a role in the pathogenesis of MAFLD through diverse pathways that impact host immunity, fructose, and fatty acid metabolism. Future research should concentrate on developing personalized intervention strategies by modifying the diet and microbiota to prevent and treat MAFLD.

## 4. Methods

### 4.1. Data Retrieval Strategy

A comprehensive search was conducted for studies published before July 2023 investigating the correlation between MAFLD and gut microbiota. The search encompassed FASTA/Q files and was executed across prominent databases, namely, the European Molecular Biology Laboratory (EMBL)’s European Bioinformatics Institute (EMBL-EBI database, link), the National Center for Biotechnology Information (NCBI database, link), and the GMrepo database (a dedicated repository for Gut Microbiota, link) [54]. Using a combination of terms in Medical Subject Headings (MeSH) and free-text words, such as “Metabolic dysfunction-associated fatty liver disease”, “non-alcoholic fatty liver”, “non-alcoholic fatty liver disease”, “NAFLD”, “MAFLD”, and “gut microbiota” or “intestinal flora”, case-control studies assessing gut microbiota in both MAFLD patients and non-MAFLD individuals were identified. All included studies were approved by institutional review boards, and participants or their family members had provided informed consent.

### 4.2. Criteria for Inclusion and Exclusion

Inclusion and exclusion criteria of MAFLD in each study were checked for selection and the studies to meet the guidelines established in “A Multisociety Delphi Consensus Statement on New Fatty Liver Disease Nomenclature” [1]. Furthermore, studies utilizing 16S rRNA gene amplicon sequencing for analyzing gut microbiota compositions were included. The characteristics of each study are presented in Table 1. MAFLD was diagnosed with imaging techniques or liver biopsy. In the included studies, the participants were aged >18 years and had elevated serum ALT concentrations that had persisted for over six months. The exclusion criteria were hepatitis infection, autoimmune liver diseases, cholangitis, gastrointestinal or liver cancers, drug-induced liver damage, genetic liver diseases, excessive alcohol (>20 g/day), recently taking antibiotics, diagnosed malignancy within five years, HIV, chronic malnutrition, or immunosuppression [1].

### 4.3. Downloading Data and Annotation of Species

The retrieval of filtered Sequence Read Archive (SRA) data was facilitated using the SRA Toolkit version 2.5.2. Subsequently, the SRA files underwent conversion into a paired-end sequencing FASTA/Q file with the accompanying quality data. The 2021.2 version of QIIME2 was used for the comprehensive 16S microbiome analysis. Demultiplexing the sequences was conducted initially, followed by applying the DADA2 plugin within QIIME2 to filter based on quality scores [54]. Representative sequences and count tables were generated, and species classifications were assigned to the sequences. This annotation process utilized a pre-trained naive Bayes classifier provided by QIIME2, and the q2-feature-classifier plugin was instrumental in executing this step. The pre-trained naive Bayes classifier was specifically trained on Greengenes (revision 13.8), where the sequences were trimmed to include only 250 bases from the V4 hypervariable region of the 16S and pre-clustered at 99% sequence identity [54]. 

### 4.4. Core Microbial Biomarker and Enterotype Analysis

Operational taxonomic units (OTUs) featuring a relative abundance of less than 0.1% were systematically eliminated to accurately identify the core microbial biomarkers associated with MAFLD. After this filtering, linear discriminant analysis effect size (LEfSe) differential analysis was conducted to unveil OTUs exhibiting significant differential abundance, using the Kruskal–Wallis tests and linear discriminant analysis (LDA) [55]. ANOVA-Like Differential Expression tool for high throughput sequencing data (ALDEx2), a tool for differential abundance analysis, was then applied to scrutinize OTU variances utilizing Bayesian methods and determine the significance utilizing Monte Carlo sampling. The eXtreme Gradient Boosting (XGBoost) machine learning method, a widely utilized gradient boosting algorithm, was chosen for its capability to refine the microbial candidates potentially contributing to MAFLD risk. To interpret the outcomes of the XGBoost model, we applied the Shapley Additive exPlanations (SHAP) method and utilized the SHAP package (version 0.39.0) to compute the average absolute SHAP values for each variable [56]. This process allowed us to observe the importance and impact of individual variables on the classification. 

Deep cross-fusion networks for genome-scale identification of pathogens (DCiPatho) for toxicity prediction of bacteria were used to assess the pathogenicity of differentially relative abundant microbes [57]. DCiPatho is a tool for predicting pathogenic microbial genes within the microbiota by analyzing gene toxicity, thus assisting in identifying potential microbial biomarkers (https://github.com/LorMeBioAI/DCiPatho, accessed on 20 September 2023). Microbiota classification and count information were utilized to categorize enterotypes with principal component analysis (PCA). The determination of enterotypes relied on ensuring that the eigenvalues exceeded 1.5, and this criterion was implemented using the “FactoMineR” and “Factoextra” packages within the R software environment.

### 4.5. Subgroup Analysis of Multiple Pathogenic Factors

We investigated the relationship between gut microbiota and age, gender, ethnicity, and obesity concerning MAFLD, categorized by enterotypes. Subsequently, redundancy analysis (RDA) was carried out using the R packages “stats” and “vegan” (version 2.5–7) to elucidate the relationship between risk factors and microbiota [58]. All the procedures above were conducted in R (version 4.1.3). Due to a small number of healthy Asians with ET-P, we conducted a posterior analysis using the Bayesian model with the “rstan” package and visualized the results with “bayesplot” to reduce the possibility of false positives. Bayesian analysis is advantageous in adapting to small sample sizes by updating probability estimates using Bayes’ theorem, thereby enhancing the accuracy and reliability of analysis in small datasets [59].

### 4.6. Analysis of α and β Diversity

Alpha diversity was determined with the Chao and Shannon indexes using the relative abundance table classification at the species level. Beta diversity was calculated by PCA using the R package stats (princomp, version 3.5.0) and the ggplot2 package (version 3.2.0) [56]. It represented the comparison of the similarities between the MAFLD and non-MAFLD groups. 

### 4.7. Construction of Microbiota Co-Occurrence Network and Functional Annotation

We utilized FastSpar (version 0.0.10), an efficient and parallel SparCC algorithm, to deduce the correlation coefficient (Rho) of the gut microbiome. *p*-values were calculated using an unbiased estimator, with screening criteria set at Rho ≥ |0.01| and a *p*-value ≤ 0.05. Co-occurrence network visualization was conducted using Gephi (version 0.9.5). Functional predictions of the gut microbiome were based on gene function predictions using Phylogenetic Investigation of Communities by Reconstruction of Unobserved States (PICRUSt2) software and the Kyoto Encyclopedia of Genes and Genomes (KEGG) database website for gene function analysis (https://www.kegg.jp/kegg/, accessed on 13 October 2023) [60]. Normalization was performed using the Z-score method, and heatmap visualization was performed using the pheatmap package (version 1.0.12). Subsequently, the association analysis between biological functions and microbial biomarkers was carried out by combining the ggplot2 package (version 3.3.3) and the linkET package (version 0.0.7.4). The Mantel test was used to assess the correlation between the two matrices. All these procedures were performed in R (version 4.1.3).

### 4.8. Statistical Analysis

Statistical analysis was conducted using SPSS software (version 13.0, SPSS, Armonk, NY, USA). The distinction between the MAFLD and non-MAFLD groups was assessed using a one-way analysis of similarities (ANOSIM) Bonferroni test to reduce the statistical errors in calculating the microbial composition between the two groups while adjusting for age and gender and to control for statistical errors that may arise from multiple comparisons. The difference between MAFLD and non-MAFLD individuals was examined according to enterotypes using the Kruskal–Wallis T-test. Statistical significance was considered as *p* < 0.05. 

## Figures and Tables

**Table 1:** The characteristics of each selected case-control study for metabolic dysfunction-associated fatty liver disease (MAFLD).

Study ID	Year	Case	Control	Country	Age	BMI	Gender (M/F)	Inclusion Criteria	Exclusion Criteria
PRJNA246121	2020	53	32	China	45.1 ± 10.3	26.4 ± 4.9	31/54	Based on guidelines by the American Gastroenterological Association, AASLD, and ACG.	Excessive alcohol drinking; HBV/HCV; autoimmune liver diseases; liver cancer or gastrointestinal diseases; recently taking antibiotics or probiotics; pregnancy or lactation.
PRJNA540738	2020	125	19	Germany	50.0 ± 14.0	31.4 ± 4.3	78/66	Daily alcohol <10 g for women and <20 g for men; ≥5% liver fat on biopsy; no steatogenic medication; no secondary hepatic fat diseases.	Antibiotics in the last six months; malignancies; pregnancy; under 18; significant weight loss or prior NASH studies involvement.
PRJEB28350	2019	77	117	USA	50.4 ± 18.5	30.0 ± 6.2	56/138	Adults ≥18; ultrasound-confirmed hepatic fat deposition; increased serum ALT levels in the past six months.	Hepatitis viruses; autoimmune hepatitis; cholangitis; gastrointestinal or liver cancers; taking medications for steatosis from drugs; Wilson’s or hemochromatosis; excessive alcohol; recently taking antibiotics; HIV; malnutrition or immunosuppression-related diseases.
PRJEB27662	2018	262	38	Korea	46.2 ± 12.1	29.0 ± 3.7	165/135	Adults aged ≥18 with ultrasound-confirmed fatty liver; high serum ALT in the last six months.	Hepatitis infection; autoimmune liver diseases; cholangitis; gastrointestinal or liver cancers; drug-induced liver damage; genetic liver diseases; excessive alcohol; recently taking antibiotics; diagnosed malignancy within five years; HIV; chronic malnutrition or immunosuppression.
PRJEB40538	2021	27	18	Italy	48.8 ± 9.36	34.4 ± 4.41	26/19	Adults aged 18–67 with NAFLD/NASH confirmed by LiverMultiScan and BMI over 30 kg/m^2^.	Hepatotoxic drugs; other liver diseases including cirrhosis or hepatitis C; untreated diabetes; thyroid issues; hypopituitarism; Cushing’s disease; alcohol and drug abuse.
PRJNA518731	2019	36	16	New Zealand	49.6 ± 11.8	31.6 ± 3.8	27/25	NAFLD histology or BMI > 27 kg/m^2^ with type 2 diabetes/metabolic syndrome and high ALT; age > 18 < 75.	Daily drinking > 20 g for the last three months in 5 years; pending or past bariatric surgery; allergies to eggs, nuts, or metronidazole; substance abuse history; eGFR < 60 mL/min or other trial participation.

Case: MAFLD; Control: non-MAFLD; BMI: Body Mass Index; M, male; F: female.

**Table 2:** Relative abundance differences at the genus level in microbial communities associated with metabolic dysfunction-associated fatty liver disease (MAFLD).

	Mean ± SD (non-MAFLD)	Mean ± SD (MAFLD)	Log2fc(MAFLD/non-MAFLD)	*p*-Value	Bonferroni *p*-Value
*Prevotella*	4.20 ± 1.14	11.46 ± 0.82	−1.447	1.14 × 10^−1^³	1.83 × 10^−12^
*Parabacteroides*	1.72 ± 0.22	3.48 ± 0.23	−1.016	6.82 × 10^−7^	1.09 × 10^−5^
*Blautia*	7.87 ± 0.84	4.82 ± 0.32	0.706	1.34 × 10^−5^	0.000215
*Dialister*	0.71 ± 0.16	2.40 ± 0.26	−1.765	1.54 × 10^−5^	0.000247
*Bacteroides*	17.70 ± 1.50	24.14 ± 0.84	−0.448	5.76 × 10^−5^	0.000922
*Streptococcus*	0.77 ± 0.14	2.09 ± 0.25	−1.446	0.0117	0.187034
*Clostridium*	3.44 ± 0.46	1.86 ± 0.11	0.884	0.0203	0.325434
*Dorea*	2.10 ± 0.20	1.55 ± 0.11	0.436	0.0250	0.401303
*Roseburia*	3.40 ± 0.45	3.13 ± 0.18	0.116	0.0358	0.572061
*Faecalibacterium*	10.08 ± 0.84	8.06 ± 0.32	0.323	0.2619	1
*Oscillospira*	3.06 ± 0.36	2.17 ± 0.14	0.496	0.3104	1
*Ruminococcus*	4.47 ± 0.44	4.27 ± 0.22	0.066	0.5831	1
*Lachnospira*	1.20 ± 0.16	1.21 ± 0.13	−0.010	0.6785	1
*Coprococcus*	3.50 ± 0.46	2.48 ± 0.15	0.498	0.7701	1
*Bifidobacterium*	5.91 ± 0.82	3.82 ± 0.31	0.628	0.9674	1
*Others*	29.88 ± 2.18	23.05 ± 0.68	0.375	0.3265	1

## Data Availability

All data generated or analyzed in the current study are available in the figshare repository (https://figshare.com/account/home, https://doi.org/10.6084/m9.figshare.24903357). There are no restrictions on any datasets used, and other researchers can access all of them.

## Supplementary Materials

The following supporting information can be downloaded at: https://www.mdpi.com/article/10.3390/ijms25042183/s1.
