# Supplementary material for: Microbial Dysbiosis Linked to Metabolic Dysfunction-Associated Fatty Liver Disease in Asians: Prevotella copri Promotes Lipopolysaccharide Biosynthesis and Network Instability in the Prevotella Enterotype
Source: Int J Mol Sci. 2024 Feb 11;25(4):2183. doi: 10.3390/ijms25042183 (PMC10889285; doi:10.3390/ijms25042183)
Supplement: Supplementary file 1 [file ijms-25-02183-s001.zip › ijms-2821487-supplementary.pdf]

Supplementary Figure S1A

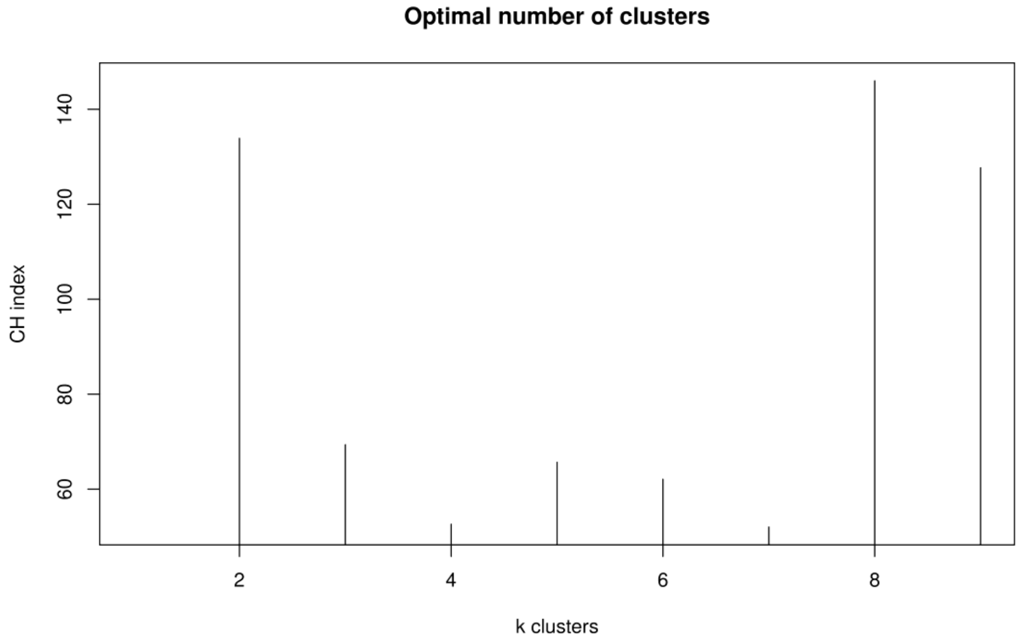

Figure S1B

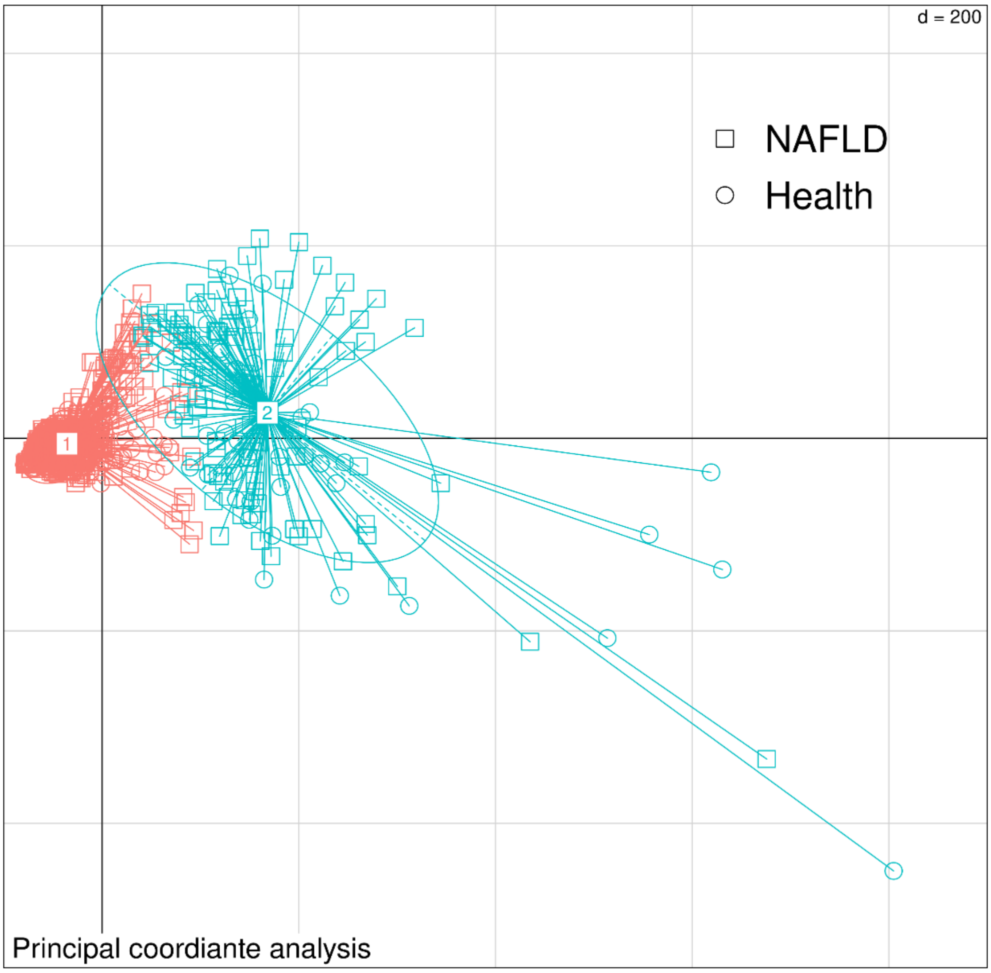

Supplementary Figure S2A

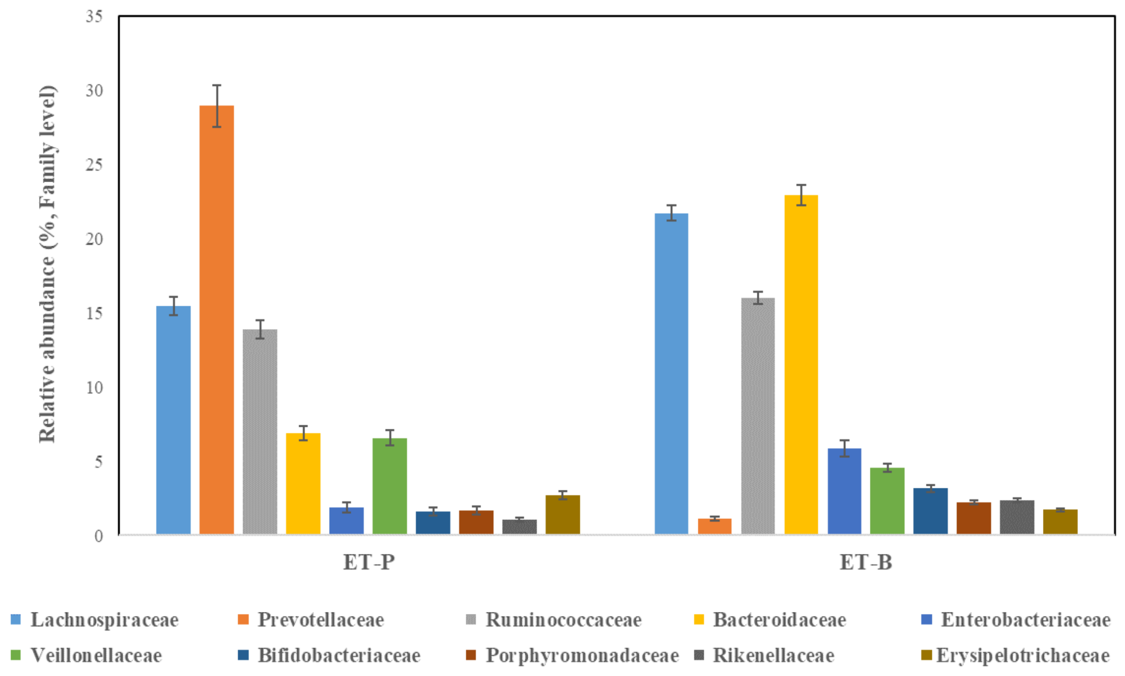

Figure S2B

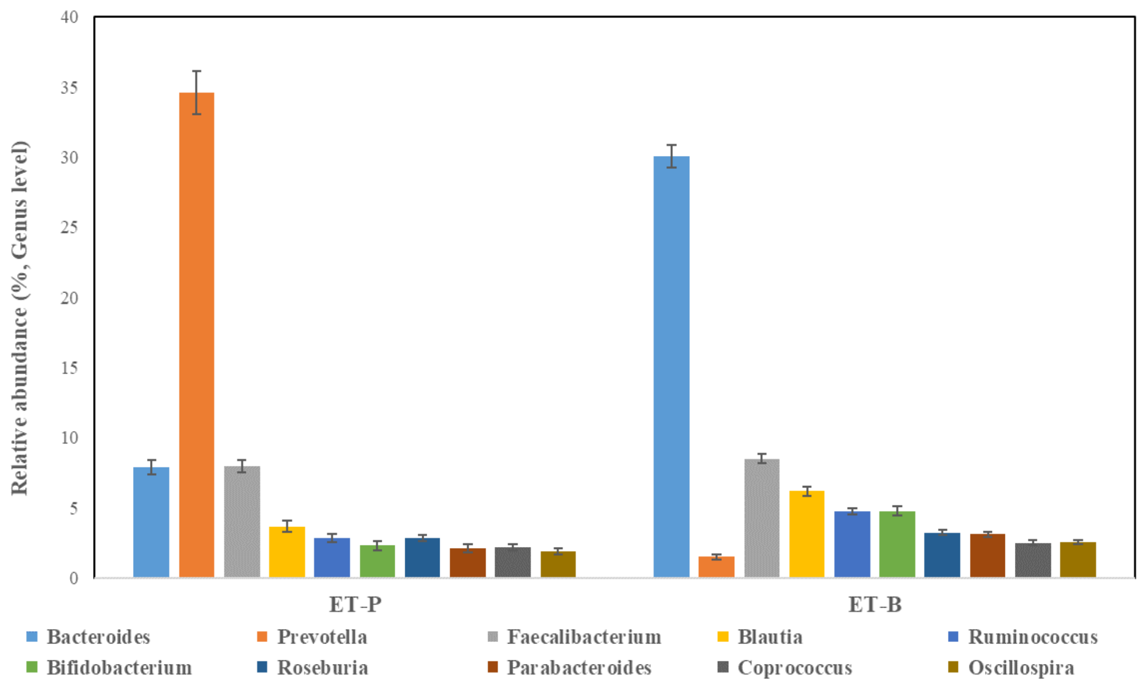

Supplementary Figure S3

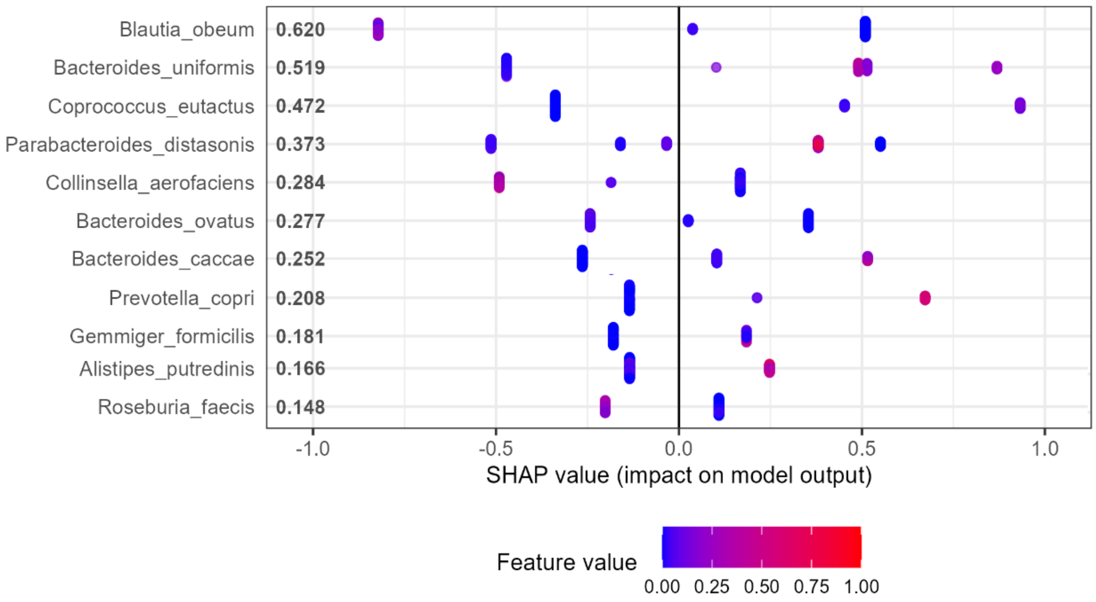

Supplementary Figure S4

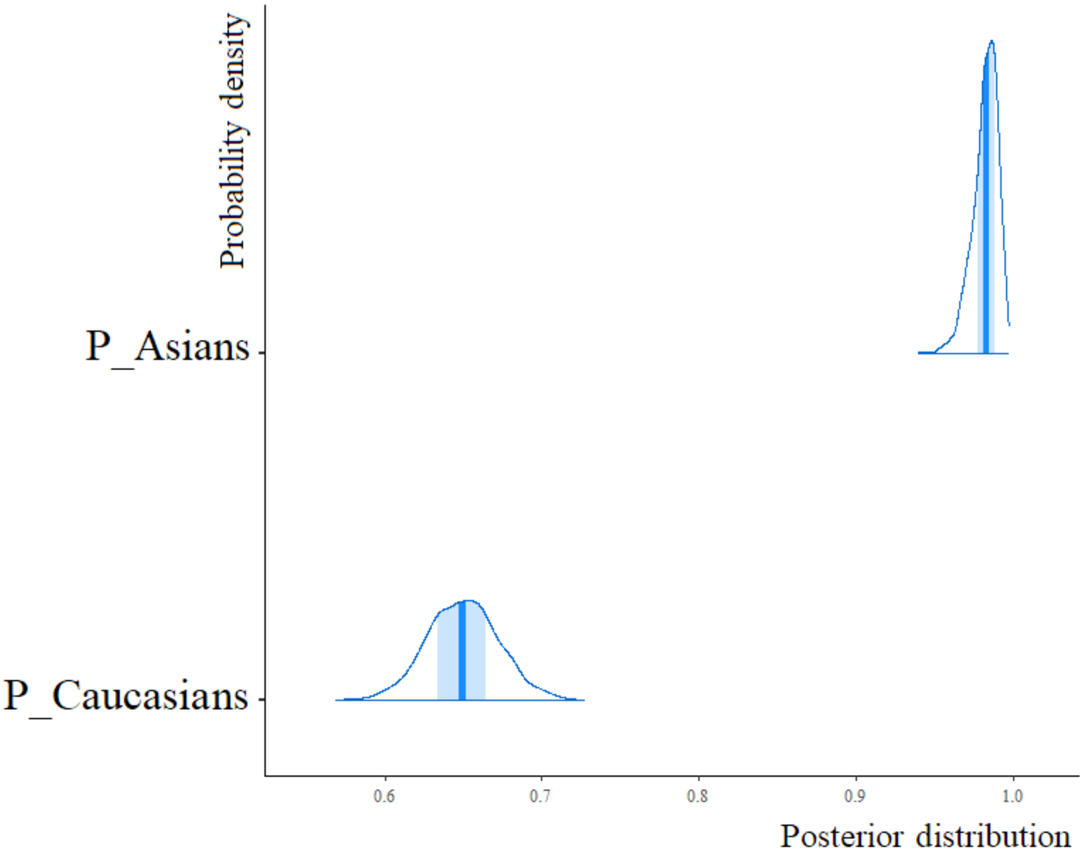

Supplementary Figure S5

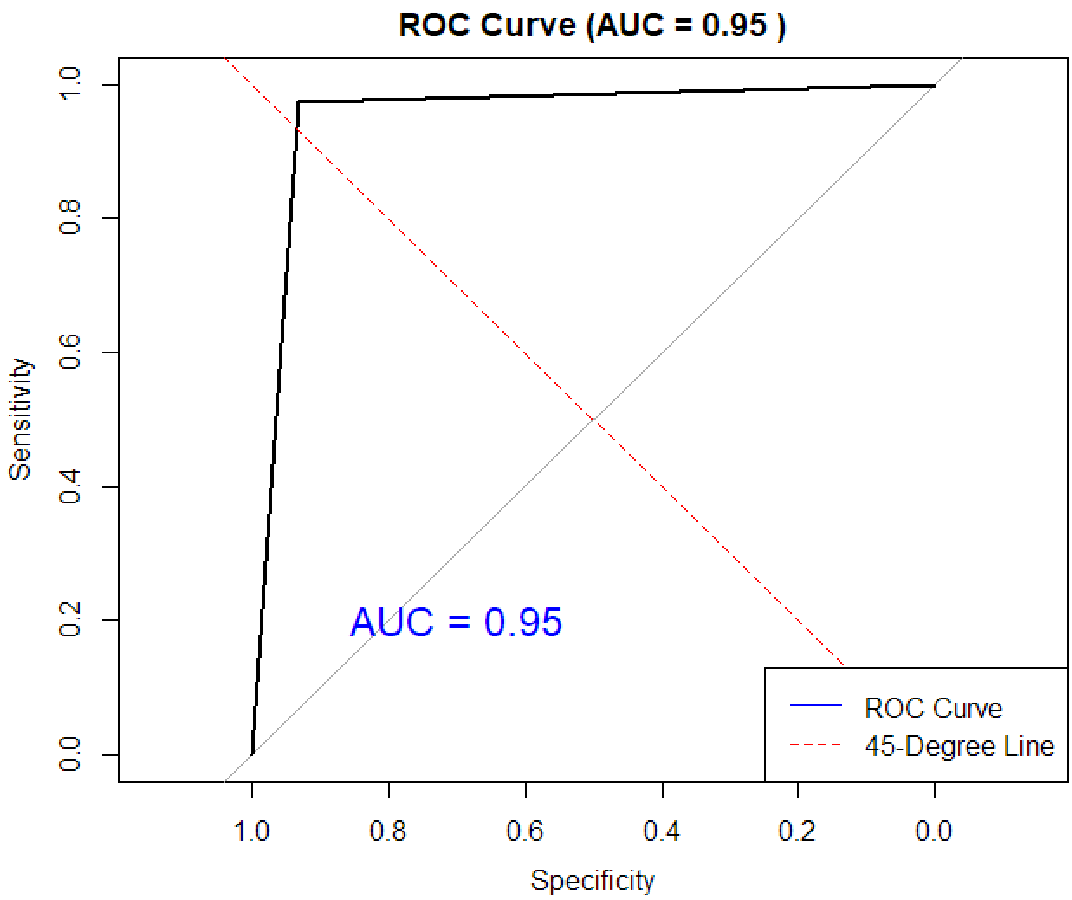

Supplementary Figure S6

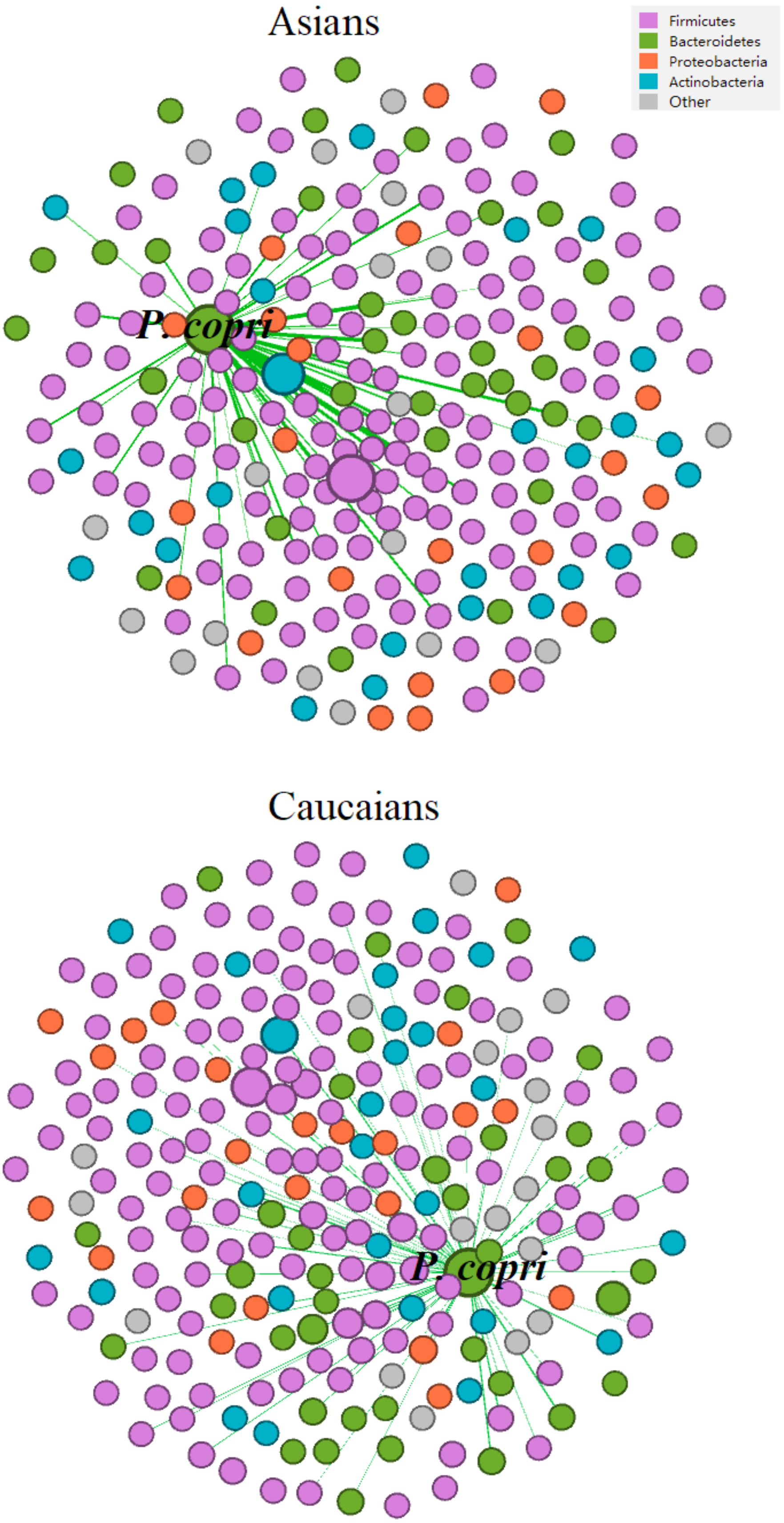

Supplementary table S1. Performance data for XGBoost machine learning.

	Specificity	Specificity	Sensitivity	ROC AUC
ET-B	0.8266667	0.7478788	0.837607	0.7455556
ET-P	0.9923664	1	0.990446	0.9890937

Supplementary table S2. Detailed clinical data for each sample.

Project number	Sample ID	Age	Gender	BMI	Country	Race	Group
PRJEB28350	ERR2753669	52	Female	38.9501	USA	Caucasian	MAFLD
PRJEB28350	ERR2753670	71	Female	31.0339	USA	Caucasian	Health
PRJEB28350	ERR2753671	63	Female	34.8643	USA	Caucasian	MAFLD
PRJEB28350	ERR2753672	63	Female	33.5616	USA	Caucasian	MAFLD
PRJEB28350	ERR2753673	61	Female	42.6692	USA	Caucasian	MAFLD
PRJEB28350	ERR2753674	40	Female	29.1277	USA	Caucasian	MAFLD
PRJEB28350	ERR2753675	41	Female	25.5556	USA	Caucasian	MAFLD
PRJEB28350	ERR2753676	47	Female	33.4223	USA	Caucasian	MAFLD
PRJEB28350	ERR2753677	64	Female	33.2671	USA	Caucasian	MAFLD
PRJEB28350	ERR2753678	42	Male	28.3685	USA	Caucasian	MAFLD
PRJEB28350	ERR2753679	37	Female	30.625	USA	Caucasian	MAFLD
PRJEB28350	ERR2753680	58	Female	26.0792	USA	Caucasian	MAFLD
PRJEB28350	ERR2753681	61	Female	25.0979	USA	Caucasian	MAFLD
PRJEB28350	ERR2753683	69	Female	30.6712	USA	Caucasian	MAFLD
PRJEB28350	ERR2753684	39	Male	30.978	USA	Caucasian	MAFLD
PRJEB28350	ERR2753685	81	Male	35.8138	USA	Caucasian	MAFLD
PRJEB28350	ERR2753686	81	Male	34.5629	USA	Caucasian	MAFLD
PRJEB28350	ERR2753687	78	Female	27.4747	USA	Caucasian	MAFLD
PRJEB28350	ERR2753689	65	Male		USA	Caucasian	MAFLD
PRJEB28350	ERR2753690	38	Male	24.2101	USA	Caucasian	Health
PRJEB28350	ERR2753691	33	Female	29.3447	USA	Caucasian	Health
PRJEB28350	ERR2753692	71	Male	31.0085	USA	Caucasian	MAFLD
PRJEB28350	ERR2753694	68	Male	28.9073	USA	Caucasian	MAFLD
PRJEB28350	ERR2753696	60	Female	26.6476	USA	Caucasian	MAFLD
PRJEB28350	ERR2753697	39	Female	56.7591	USA	Caucasian	Health
PRJEB28350	ERR2753698	30	Female	27.4004	USA	Caucasian	MAFLD
PRJEB28350	ERR2753699	28	Male	34.8027	USA	Caucasian	Health
PRJEB28350	ERR2753700	66	Female	37.9188	USA	Caucasian	MAFLD
PRJEB28350	ERR2753701	43	Female	43.2341	USA	Caucasian	MAFLD
PRJEB28350	ERR2753702	33	Male	103.138	USA	Caucasian	Health
PRJEB28350	ERR2753703	45	Female	48.2878	USA	Caucasian	MAFLD
PRJEB28350	ERR2753704	42	Female	38.6396	USA	Caucasian	MAFLD
PRJEB28350	ERR2753705	66	Female	22.1631	USA	Caucasian	MAFLD
PRJEB28350	ERR2753706	63	Female	31.0735	USA	Caucasian	Health
PRJEB28350	ERR2753707	59	Female	28.4878	USA	Caucasian	MAFLD
PRJEB28350	ERR2753708	59	Female	37.2945	USA	Caucasian	MAFLD
PRJEB28350	ERR2753709	66	Female	29.8328	USA	Caucasian	MAFLD
PRJEB28350	ERR2753710	61	Female	37.5828	USA	Caucasian	MAFLD
PRJEB28350	ERR2753711	61	Female	33.011	USA	Caucasian	MAFLD
PRJEB28350	ERR2753712	73	Female	37.6636	USA	Caucasian	MAFLD
PRJEB28350	ERR2753713	71	Male	39.3951	USA	Caucasian	MAFLD
PRJEB28350	ERR2753714	64	Female	49.8215	USA	Caucasian	MAFLD
PRJEB28350	ERR2753715	79	Female	22.9767	USA	Caucasian	MAFLD

PRJEB28350	ERR2753716	47	Female	26.9788	USA	Caucasian	Health
PRJEB28350	ERR2753717	72	Female	23.8684	USA	Caucasian	MAFLD
PRJEB28350	ERR2753718	44	Female	23.7007	USA	Caucasian	Health
PRJEB28350	ERR2753719	68	Male	38.8559	USA	Caucasian	MAFLD
PRJEB28350	ERR2753720	29	Male	27.8531	USA	Caucasian	Health
PRJEB28350	ERR2753721	72	Female	71.2938	USA	Caucasian	MAFLD
PRJEB28350	ERR2753722	44	Female	40.1163	USA	Caucasian	MAFLD
PRJEB28350	ERR2753723	41	Female	38.4183	USA	Caucasian	MAFLD
PRJEB28350	ERR2753724	58	Female	45.4668	USA	Caucasian	MAFLD
PRJEB28350	ERR2753725	27	Female	33.9427	USA	Caucasian	Health
PRJEB28350	ERR2753726	79	Female	28.1221	USA	Caucasian	MAFLD
PRJEB28350	ERR2753727	60	Female	30.2545	USA	Caucasian	Health
PRJEB28350	ERR2753728	49	Female	27.9904	USA	Caucasian	MAFLD
PRJEB28350	ERR2753729	56	Female	41.8733	USA	Caucasian	MAFLD
PRJEB28350	ERR2753730	69	Female	25.0942	USA	Caucasian	MAFLD
PRJEB28350	ERR2753731	30	Female	36.2053	USA	Caucasian	MAFLD
PRJEB28350	ERR2753732	73	Female	28.7	USA	Caucasian	MAFLD
PRJEB28350	ERR2753733	71	Female	39.5	USA	Caucasian	MAFLD
PRJEB28350	ERR2753734	18	Female	20.5	USA	Caucasian	Health
PRJEB28350	ERR2753735	18	Female	21.2	USA	Caucasian	Health
PRJEB28350	ERR2753736	25	Male	17.8	USA	Caucasian	Health
PRJEB28350	ERR2753737	25	Male	18.8	USA	Caucasian	Health
PRJEB28350	ERR2753738	22	Female	19.8	USA	Caucasian	Health
PRJEB28350	ERR2753739	22	Female	21.3	USA	Caucasian	Health
PRJEB28350	ERR2753740	58	Male	32.6	USA	Caucasian	MAFLD
PRJEB28350	ERR2753741	58	Male	32.2	USA	Caucasian	MAFLD
PRJEB28350	ERR2753742	64	Female	23.4	USA	Caucasian	Health
PRJEB28350	ERR2753743	64	Female	21.9	USA	Caucasian	Health
PRJEB28350	ERR2753744	66	Female	22.2	USA	Caucasian	Health
PRJEB28350	ERR2753745	66	Female	23.4	USA	Caucasian	Health
PRJEB28350	ERR2753746	21	Female	20.5	USA	Caucasian	Health
PRJEB28350	ERR2753747	71	Female	24.6	USA	Caucasian	Health
PRJEB28350	ERR2753748	71	Female	23.9	USA	Caucasian	Health
PRJEB28350	ERR2753749	82	Female	22.1	USA	Caucasian	MAFLD
PRJEB28350	ERR2753751	51	Female	29.6	USA	Caucasian	Health
PRJEB28350	ERR2753752	84	Female	23.6	USA	Caucasian	Health
PRJEB28350	ERR2753753	84	Female	25.3	USA	Caucasian	Health
PRJEB28350	ERR2753754	51	Female	25.8	USA	Caucasian	Health
PRJEB28350	ERR2753755	19	Female	29.5	USA	Caucasian	Health
PRJEB28350	ERR2753756	61	Female	29.1	USA	Caucasian	MAFLD
PRJEB28350	ERR2753757	61	Female	28.8	USA	Caucasian	MAFLD
PRJEB28350	ERR2753758	20	Male	25	USA	Caucasian	Health
PRJEB28350	ERR2753759	21	Male	29	USA	Caucasian	Health
PRJEB28350	ERR2753760	72	Female	24.6	USA	Caucasian	Health
PRJEB28350	ERR2753761	72	Female	22.7	USA	Caucasian	Health
PRJEB28350	ERR2753762	56	Female	20.4	USA	Caucasian	Health

PRJEB28350	ERR2753763	23	Male	26.3	USA	Caucasian	Health
PRJEB28350	ERR2753764	55	Female	30	USA	Caucasian	Health
PRJEB28350	ERR2753765	19	Male	17.9	USA	Caucasian	Health
PRJEB28350	ERR2753766	52	Male	32.6	USA	Caucasian	MAFLD
PRJEB28350	ERR2753767	51	Male	31.8	USA	Caucasian	MAFLD
PRJEB28350	ERR2753768	32	Female	43.9	USA	Caucasian	Health
PRJEB28350	ERR2753769	32	Female	36.8	USA	Caucasian	MAFLD
PRJEB28350	ERR2753770	25	Female	22.2	USA	Caucasian	Health
PRJEB28350	ERR2753771	29	Female	21.2	USA	Caucasian	Health
PRJEB28350	ERR2753772	21	Female	24.1	USA	Caucasian	Health
PRJEB28350	ERR2753773	22	Female	19.4	USA	Caucasian	Health
PRJEB28350	ERR2753774	51	Female	27.7	USA	Caucasian	Health
PRJEB28350	ERR2753775	18	Female	23.3	USA	Caucasian	Health
PRJEB28350	ERR2753776	23	Female	21.8	USA	Caucasian	Health
PRJEB28350	ERR2753777	21	Male	19.2	USA	Caucasian	Health
PRJEB28350	ERR2753778	60	Female	26.6	USA	Caucasian	Health
PRJEB28350	ERR2753779	60	Female	24.9	USA	Caucasian	Health
PRJEB28350	ERR2753780	64	Female	22.6	USA	Caucasian	Health
PRJEB28350	ERR2753781	64	Female	21.9	USA	Caucasian	MAFLD
PRJEB28350	ERR2753783	21	Male	23.5	USA	Caucasian	Health
PRJEB28350	ERR2753784	56	Female	25.4	USA	Caucasian	Health
PRJEB28350	ERR2753785	22	Male	29.8	USA	Caucasian	Health
PRJEB28350	ERR2753786	49	Male	36.9	USA	Caucasian	MAFLD
PRJEB28350	ERR2753787	49	Male	36	USA	Caucasian	MAFLD
PRJEB28350	ERR2753788	64	Female	27.3	USA	Caucasian	Health
PRJEB28350	ERR2753789	64	Female	22.8	USA	Caucasian	Health
PRJEB28350	ERR2753790	22	Male	30.3	USA	Caucasian	Health
PRJEB28350	ERR2753791	22	Male	28.8	USA	Caucasian	Health
PRJEB28350	ERR2753792	49	Female	25.4	USA	Caucasian	MAFLD
PRJEB28350	ERR2753793	49	Female	26	USA	Caucasian	MAFLD
PRJEB28350	ERR2753794	63	Female	25.4	USA	Caucasian	Health
PRJEB28350	ERR2753795	22	Female	23.7	USA	Caucasian	Health
PRJEB28350	ERR2753796	30	Male	21.4	USA	Caucasian	Health
PRJEB28350	ERR2753797	30	Male	23.3	USA	Caucasian	Health
PRJEB28350	ERR2753798	52	Female	21.9	USA	Caucasian	Health
PRJEB28350	ERR2753799	52	Female	24.1	USA	Caucasian	Health
PRJEB28350	ERR2753800	61	Male	44.4	USA	Caucasian	Health
PRJEB28350	ERR2753801	61	Male	36.4	USA	Caucasian	Health
PRJEB28350	ERR2753802	28	Male	24.8	USA	Caucasian	Health
PRJEB28350	ERR2753803	28	Male	30.7	USA	Caucasian	Health
PRJEB28350	ERR2753804	55	Female	28.8	USA	Caucasian	Health
PRJEB28350	ERR2753805	27	Female	27.3	USA	Caucasian	Health
PRJEB28350	ERR2753806	61	Male	33.1	USA	Caucasian	MAFLD
PRJEB28350	ERR2753807	61	Male	32.1	USA	Caucasian	MAFLD
PRJEB28350	ERR2753808	74	Female	29.5	USA	Caucasian	MAFLD
PRJEB28350	ERR2753809	74	Female	23.5	USA	Caucasian	Health

PRJEB28350	ERR2753810	62	Female	38.7	USA	Caucasian	MAFLD
PRJEB28350	ERR2753811	41	Male	41.8	USA	Caucasian	Health
PRJEB28350	ERR2753812	64	Male	25	USA	Caucasian	Health
PRJEB28350	ERR2753813	27	Female	22.9	USA	Caucasian	Health
PRJEB28350	ERR2753814	27	Female	20.9	USA	Caucasian	Health
PRJEB28350	ERR2753815	33	Female	21.5	USA	Caucasian	Health
PRJEB28350	ERR2753816	33	Female	23.7	USA	Caucasian	Health
PRJEB28350	ERR2753817	24	Male	31.4	USA	Caucasian	MAFLD
PRJEB28350	ERR2753818	24	Male	29.8	USA	Caucasian	Health
PRJEB28350	ERR2753819	78	Male	36.1	USA	Caucasian	MAFLD
PRJEB28350	ERR2753820	78	Male	34	USA	Caucasian	MAFLD
PRJEB28350	ERR2753822	47	Female	42.6	USA	Caucasian	MAFLD
PRJEB28350	ERR2753823	54	Male	26.4	USA	Caucasian	Health
PRJEB28350	ERR2753824	54	Male	27.9	USA	Caucasian	Health
PRJEB28350	ERR2753825	55	Female	34.4	USA	Caucasian	Health
PRJEB28350	ERR2753826	55	Female	31.2	USA	Caucasian	Health
PRJEB28350	ERR2753827	49	Female	24	USA	Caucasian	MAFLD
PRJEB28350	ERR2753828	49	Female	20.8	USA	Caucasian	Health
PRJEB28350	ERR2753829	25	Female	20.2	USA	Caucasian	Health
PRJEB28350	ERR2753830	71	Female	25.2	USA	Caucasian	Health
PRJEB28350	ERR2753831	71	Female	27.4	USA	Caucasian	Health
PRJEB28350	ERR2753832	56	Female	38.7	USA	Caucasian	MAFLD
PRJEB28350	ERR2753833	75	Female	21.1	USA	Caucasian	Health
PRJEB28350	ERR2753834	19	Female	28.7	USA	Caucasian	Health
PRJEB28350	ERR2753835	19	Male	37	USA	Caucasian	Health
PRJEB28350	ERR2753836	51	Male	29.8	USA	Caucasian	MAFLD
PRJEB28350	ERR2753838	55	Female	33.1	USA	Caucasian	Health
PRJEB28350	ERR2753839	58	Male	25.2	USA	Caucasian	Health
PRJEB28350	ERR2753840	32	Male	21.6	USA	Caucasian	MAFLD
PRJEB28350	ERR2753841	53	Female	23.1	USA	Caucasian	Health
PRJEB28350	ERR2753842	56	Female	22	USA	Caucasian	Health
PRJEB28350	ERR2753843	49	Female	38.4	USA	Caucasian	MAFLD
PRJEB28350	ERR2753844	79	Female	26.4	USA	Caucasian	MAFLD
PRJEB28350	ERR2753845	65	Female	20.6	USA	Caucasian	Health
PRJEB28350	ERR2753846	65	Female	19.6	USA	Caucasian	Health
PRJEB28350	ERR2753847	65	Female	24.7	USA	Caucasian	Health
PRJEB28350	ERR2753848	60	Female		USA	Caucasian	Health
PRJEB28350	ERR2753849	60	Female	27	USA	Caucasian	MAFLD
PRJEB28350	ERR2753850	71	Female	34.8	USA	Caucasian	Health
PRJEB28350	ERR2753851	39	Female	23.5	USA	Caucasian	Health
PRJEB28350	ERR2753852	27	Female	28.7	USA	Caucasian	Health
PRJEB28350	ERR2753853	27	Female	29.3	USA	Caucasian	Health
PRJEB28350	ERR2753854	43	Female	39.4	USA	Caucasian	Health
PRJEB28350	ERR2753856	29	Female	20.6	USA	Caucasian	Health
PRJEB28350	ERR2753857	67	Female	20.3	USA	Caucasian	Health
PRJEB28350	ERR2753858	67	Female	22.1	USA	Caucasian	Health

PRJEB28350	ERR2753859	63	Female	23.6	USA	Caucasian	Health
PRJEB28350	ERR2753860	63	Female	24.9	USA	Caucasian	Health
PRJEB28350	ERR2753861	64	Female	25.9	USA	Caucasian	Health
PRJEB28350	ERR2753862	64	Female	32.3	USA	Caucasian	Health
PRJEB28350	ERR2753863	62	Male	33.7	USA	Caucasian	Health
PRJEB28350	ERR2753864	62	Female	27.8	USA	Caucasian	Health
PRJEB28350	ERR2753865	21	Male	20.8	USA	Caucasian	Health
PRJEB28350	ERR2753866	58	Male	57.6	USA	Caucasian	Health
PRJEB28350	ERR2753867	65	Female	32.5	USA	Caucasian	Health
PRJEB28350	ERR2753868	65	Female	30.1	USA	Caucasian	Health
PRJEB28350	ERR2753869	21	Male	24.4	USA	Caucasian	Health
PRJEB28350	ERR2753870	21	Male	21.9	USA	Caucasian	Health
PRJEB28350	ERR2753871	56	Male	27.1	USA	Caucasian	Health
PRJEB27662	ERR4135132				Korea	Asian	Health
PRJEB27662	ERR4135133				Korea	Asian	Health
PRJEB27662	ERR4135134				Korea	Asian	Health
PRJEB27662	ERR4135135				Korea	Asian	Health
PRJEB27662	ERR4135136				Korea	Asian	Health
PRJEB27662	ERR4135137				Korea	Asian	Health
PRJEB27662	ERR4135138				Korea	Asian	Health
PRJEB27662	ERR4135139				Korea	Asian	Health
PRJEB27662	ERR4135140				Korea	Asian	Health
PRJEB27662	ERR4135141				Korea	Asian	Health
PRJEB27662	ERR4135142				Korea	Asian	Health
PRJEB27662	ERR4135143				Korea	Asian	Health
PRJEB27662	ERR4135144				Korea	Asian	Health
PRJEB27662	ERR4135145				Korea	Asian	Health
PRJEB27662	ERR4135146				Korea	Asian	Health
PRJEB27662	ERR4135147				Korea	Asian	Health
PRJEB27662	ERR4135148				Korea	Asian	Health
PRJEB27662	ERR4135149				Korea	Asian	Health
PRJEB27662	ERR4135150				Korea	Asian	Health
PRJEB27662	ERR4135151				Korea	Asian	Health
PRJEB27662	ERR4135152				Korea	Asian	Health
PRJEB27662	ERR4135153				Korea	Asian	Health
PRJEB27662	ERR4135154				Korea	Asian	Health
PRJEB27662	ERR4135155				Korea	Asian	Health
PRJEB27662	ERR4135156				Korea	Asian	Health
PRJEB27662	ERR4135157				Korea	Asian	Health
PRJEB27662	ERR4135158				Korea	Asian	Health
PRJEB27662	ERR4135159				Korea	Asian	Health
PRJEB27662	ERR4135160				Korea	Asian	Health
PRJEB27662	ERR4135161				Korea	Asian	Health
PRJEB27662	ERR4135162				Korea	Asian	Health
PRJEB27662	ERR4135163				Korea	Asian	Health
PRJEB27662	ERR4135164				Korea	Asian	Health

PRJEB27662	ERR4135165		Korea	Asian	Health
PRJEB27662	ERR4135166		Korea	Asian	Health
PRJEB27662	ERR4135167		Korea	Asian	Health
PRJEB27662	ERR4135168		Korea	Asian	Health
PRJEB27662	ERR4135169		Korea	Asian	Health
PRJEB27662	ERR4135170		Korea	Asian	MAFLD
PRJEB27662	ERR4135171		Korea	Asian	MAFLD
PRJEB27662	ERR4135172		Korea	Asian	MAFLD
PRJEB27662	ERR4135173		Korea	Asian	MAFLD
PRJEB27662	ERR4135174		Korea	Asian	MAFLD
PRJEB27662	ERR4135175		Korea	Asian	MAFLD
PRJEB27662	ERR4135176		Korea	Asian	MAFLD
PRJEB27662	ERR4135177		Korea	Asian	MAFLD
PRJEB27662	ERR4135178		Korea	Asian	MAFLD
PRJEB27662	ERR4135179		Korea	Asian	MAFLD
PRJEB27662	ERR4135180		Korea	Asian	MAFLD
PRJEB27662	ERR4135181		Korea	Asian	MAFLD
PRJEB27662	ERR4135182		Korea	Asian	MAFLD
PRJEB27662	ERR4135183		Korea	Asian	MAFLD
PRJEB27662	ERR4135184		Korea	Asian	MAFLD
PRJEB27662	ERR4135185		Korea	Asian	MAFLD
PRJEB27662	ERR4135186		Korea	Asian	MAFLD
PRJEB27662	ERR4135187		Korea	Asian	MAFLD
PRJEB27662	ERR4135188		Korea	Asian	MAFLD
PRJEB27662	ERR4135189		Korea	Asian	MAFLD
PRJEB27662	ERR4135190		Korea	Asian	MAFLD
PRJEB27662	ERR4135191		Korea	Asian	MAFLD
PRJEB27662	ERR4135192		Korea	Asian	MAFLD
PRJEB27662	ERR4135193		Korea	Asian	MAFLD
PRJEB27662	ERR4135194		Korea	Asian	MAFLD
PRJEB27662	ERR4135195		Korea	Asian	MAFLD
PRJEB27662	ERR4135196		Korea	Asian	MAFLD
PRJEB27662	ERR4135197		Korea	Asian	MAFLD
PRJEB27662	ERR4135198		Korea	Asian	MAFLD
PRJEB27662	ERR4135199		Korea	Asian	MAFLD
PRJEB27662	ERR4135200		Korea	Asian	MAFLD
PRJEB27662	ERR4135201		Korea	Asian	MAFLD
PRJEB27662	ERR4135202		Korea	Asian	MAFLD
PRJEB27662	ERR4135203		Korea	Asian	MAFLD
PRJEB27662	ERR4135204		Korea	Asian	MAFLD
PRJEB27662	ERR4135205		Korea	Asian	MAFLD
PRJEB27662	ERR4135206		Korea	Asian	MAFLD
PRJEB27662	ERR4135207		Korea	Asian	MAFLD
PRJEB27662	ERR4135208		Korea	Asian	MAFLD
PRJEB27662	ERR4135209		Korea	Asian	MAFLD
PRJEB27662	ERR4135210		Korea	Asian	MAFLD

PRJEB27662	ERR4135211		Korea	Asian	MAFLD
PRJEB27662	ERR4135212		Korea	Asian	MAFLD
PRJEB27662	ERR4135213		Korea	Asian	MAFLD
PRJEB27662	ERR4135214		Korea	Asian	MAFLD
PRJEB27662	ERR4135215		Korea	Asian	MAFLD
PRJEB27662	ERR4135216		Korea	Asian	MAFLD
PRJEB27662	ERR4135217		Korea	Asian	MAFLD
PRJEB27662	ERR4135218		Korea	Asian	MAFLD
PRJEB27662	ERR4135219		Korea	Asian	MAFLD
PRJEB27662	ERR4135220		Korea	Asian	MAFLD
PRJEB27662	ERR4135221		Korea	Asian	MAFLD
PRJEB27662	ERR4135222		Korea	Asian	MAFLD
PRJEB27662	ERR4135223		Korea	Asian	MAFLD
PRJEB27662	ERR4135224		Korea	Asian	MAFLD
PRJEB27662	ERR4135225		Korea	Asian	MAFLD
PRJEB27662	ERR4135226		Korea	Asian	MAFLD
PRJEB27662	ERR4135227		Korea	Asian	MAFLD
PRJEB27662	ERR4135228		Korea	Asian	MAFLD
PRJEB27662	ERR4135229		Korea	Asian	MAFLD
PRJEB27662	ERR4135230		Korea	Asian	MAFLD
PRJEB27662	ERR4135231		Korea	Asian	MAFLD
PRJEB27662	ERR4135232		Korea	Asian	MAFLD
PRJEB27662	ERR4135233		Korea	Asian	MAFLD
PRJEB27662	ERR4135234		Korea	Asian	MAFLD
PRJEB27662	ERR4135235		Korea	Asian	MAFLD
PRJEB27662	ERR4135236		Korea	Asian	MAFLD
PRJEB27662	ERR4135237		Korea	Asian	MAFLD
PRJEB27662	ERR4135238		Korea	Asian	MAFLD
PRJEB27662	ERR4135239		Korea	Asian	MAFLD
PRJEB27662	ERR4135240		Korea	Asian	MAFLD
PRJEB27662	ERR4135241		Korea	Asian	MAFLD
PRJEB27662	ERR4135242		Korea	Asian	MAFLD
PRJEB27662	ERR4135243		Korea	Asian	MAFLD
PRJEB27662	ERR4135244		Korea	Asian	MAFLD
PRJEB27662	ERR4135245		Korea	Asian	MAFLD
PRJEB27662	ERR4135246		Korea	Asian	MAFLD
PRJEB27662	ERR4135247		Korea	Asian	MAFLD
PRJEB27662	ERR4135248		Korea	Asian	MAFLD
PRJEB27662	ERR4135249		Korea	Asian	MAFLD
PRJEB27662	ERR4135250		Korea	Asian	MAFLD
PRJEB27662	ERR4135251		Korea	Asian	MAFLD
PRJEB27662	ERR4135252		Korea	Asian	MAFLD
PRJEB27662	ERR4135253		Korea	Asian	MAFLD
PRJEB27662	ERR4135254		Korea	Asian	MAFLD
PRJEB27662	ERR4135255		Korea	Asian	MAFLD
PRJEB27662	ERR4135256		Korea	Asian	MAFLD

PRJEB27662	ERR4135257	Korea	Asian	MAFLD
PRJEB27662	ERR4135258	Korea	Asian	MAFLD
PRJEB27662	ERR4135259	Korea	Asian	MAFLD
PRJEB27662	ERR4135260	Korea	Asian	MAFLD
PRJEB27662	ERR4135261	Korea	Asian	MAFLD
PRJEB27662	ERR4135262	Korea	Asian	MAFLD
PRJEB27662	ERR4135263	Korea	Asian	MAFLD
PRJEB27662	ERR4135264	Korea	Asian	MAFLD
PRJEB27662	ERR4135265	Korea	Asian	MAFLD
PRJEB27662	ERR4135266	Korea	Asian	MAFLD
PRJEB27662	ERR4135267	Korea	Asian	MAFLD
PRJEB27662	ERR4135268	Korea	Asian	MAFLD
PRJEB27662	ERR4135269	Korea	Asian	MAFLD
PRJEB27662	ERR4135270	Korea	Asian	MAFLD
PRJEB27662	ERR4135271	Korea	Asian	MAFLD
PRJEB27662	ERR4135272	Korea	Asian	MAFLD
PRJEB27662	ERR4135273	Korea	Asian	MAFLD
PRJEB27662	ERR4135274	Korea	Asian	MAFLD
PRJEB27662	ERR4135275	Korea	Asian	MAFLD
PRJEB27662	ERR4135276	Korea	Asian	MAFLD
PRJEB27662	ERR4135277	Korea	Asian	MAFLD
PRJEB27662	ERR4135278	Korea	Asian	MAFLD
PRJEB27662	ERR4135279	Korea	Asian	MAFLD
PRJEB27662	ERR4135280	Korea	Asian	MAFLD
PRJEB27662	ERR4135281	Korea	Asian	MAFLD
PRJEB27662	ERR4135282	Korea	Asian	MAFLD
PRJEB27662	ERR4135283	Korea	Asian	MAFLD
PRJEB27662	ERR4135284	Korea	Asian	MAFLD
PRJEB27662	ERR4135285	Korea	Asian	MAFLD
PRJEB27662	ERR4135286	Korea	Asian	MAFLD
PRJEB27662	ERR4135287	Korea	Asian	MAFLD
PRJEB27662	ERR4135288	Korea	Asian	MAFLD
PRJEB27662	ERR4135289	Korea	Asian	MAFLD
PRJEB27662	ERR4135290	Korea	Asian	MAFLD
PRJEB27662	ERR4135291	Korea	Asian	MAFLD
PRJEB27662	ERR4135292	Korea	Asian	MAFLD
PRJEB27662	ERR4135293	Korea	Asian	MAFLD
PRJEB27662	ERR4135294	Korea	Asian	MAFLD
PRJEB27662	ERR4135295	Korea	Asian	MAFLD
PRJEB27662	ERR4135296	Korea	Asian	MAFLD
PRJEB27662	ERR4135297	Korea	Asian	MAFLD
PRJEB27662	ERR4135298	Korea	Asian	MAFLD
PRJEB27662	ERR4135299	Korea	Asian	MAFLD
PRJEB27662	ERR4135300	Korea	Asian	MAFLD
PRJEB27662	ERR4135301	Korea	Asian	MAFLD
PRJEB27662	ERR4135302	Korea	Asian	MAFLD

PRJEB27662	ERR4135303	Korea	Asian	MAFLD
PRJEB27662	ERR4135304	Korea	Asian	MAFLD
PRJEB27662	ERR4135305	Korea	Asian	MAFLD
PRJEB27662	ERR4135306	Korea	Asian	MAFLD
PRJEB27662	ERR4135307	Korea	Asian	MAFLD
PRJEB27662	ERR4135308	Korea	Asian	MAFLD
PRJEB27662	ERR4135309	Korea	Asian	MAFLD
PRJEB27662	ERR4135310	Korea	Asian	MAFLD
PRJEB27662	ERR4135311	Korea	Asian	MAFLD
PRJEB27662	ERR4135312	Korea	Asian	MAFLD
PRJEB27662	ERR4135313	Korea	Asian	MAFLD
PRJEB27662	ERR4135314	Korea	Asian	MAFLD
PRJEB27662	ERR4135315	Korea	Asian	MAFLD
PRJEB27662	ERR4135316	Korea	Asian	MAFLD
PRJEB27662	ERR4135317	Korea	Asian	MAFLD
PRJEB27662	ERR4135318	Korea	Asian	MAFLD
PRJEB27662	ERR4135319	Korea	Asian	MAFLD
PRJEB27662	ERR4135320	Korea	Asian	MAFLD
PRJEB27662	ERR4135321	Korea	Asian	MAFLD
PRJEB27662	ERR4135322	Korea	Asian	MAFLD
PRJEB27662	ERR4135323	Korea	Asian	MAFLD
PRJEB27662	ERR4135324	Korea	Asian	MAFLD
PRJEB27662	ERR4135325	Korea	Asian	MAFLD
PRJEB27662	ERR4135326	Korea	Asian	MAFLD
PRJEB27662	ERR4135327	Korea	Asian	MAFLD
PRJEB27662	ERR4135328	Korea	Asian	MAFLD
PRJEB27662	ERR4135329	Korea	Asian	MAFLD
PRJEB27662	ERR4135330	Korea	Asian	MAFLD
PRJEB27662	ERR4135331	Korea	Asian	MAFLD
PRJEB27662	ERR4135332	Korea	Asian	MAFLD
PRJEB27662	ERR4135333	Korea	Asian	MAFLD
PRJEB27662	ERR4135334	Korea	Asian	MAFLD
PRJEB27662	ERR4135335	Korea	Asian	MAFLD
PRJEB27662	ERR4135336	Korea	Asian	MAFLD
PRJEB27662	ERR4135337	Korea	Asian	MAFLD
PRJEB27662	ERR4135338	Korea	Asian	MAFLD
PRJEB27662	ERR4135339	Korea	Asian	MAFLD
PRJEB27662	ERR4135340	Korea	Asian	MAFLD
PRJEB27662	ERR4135341	Korea	Asian	MAFLD
PRJEB27662	ERR4135342	Korea	Asian	MAFLD
PRJEB27662	ERR4135343	Korea	Asian	MAFLD
PRJEB27662	ERR4135344	Korea	Asian	MAFLD
PRJEB27662	ERR4135345	Korea	Asian	MAFLD
PRJEB27662	ERR4135346	Korea	Asian	MAFLD
PRJEB27662	ERR4135347	Korea	Asian	MAFLD
PRJEB27662	ERR4135348	Korea	Asian	MAFLD

PRJEB27662	ERR4135349		Korea	Asian	MAFLD
PRJEB27662	ERR4135350		Korea	Asian	MAFLD
PRJEB27662	ERR4135351		Korea	Asian	MAFLD
PRJEB27662	ERR4135352		Korea	Asian	MAFLD
PRJEB27662	ERR4135353		Korea	Asian	MAFLD
PRJEB27662	ERR4135354		Korea	Asian	MAFLD
PRJEB27662	ERR4135355		Korea	Asian	MAFLD
PRJEB27662	ERR4135356		Korea	Asian	MAFLD
PRJEB27662	ERR4135357		Korea	Asian	MAFLD
PRJEB27662	ERR4135358		Korea	Asian	MAFLD
PRJEB27662	ERR4135359		Korea	Asian	MAFLD
PRJEB27662	ERR4135360		Korea	Asian	MAFLD
PRJEB27662	ERR4135361		Korea	Asian	MAFLD
PRJEB27662	ERR4135362		Korea	Asian	MAFLD
PRJEB27662	ERR4135363		Korea	Asian	MAFLD
PRJEB27662	ERR4135364		Korea	Asian	MAFLD
PRJEB27662	ERR4135365		Korea	Asian	MAFLD
PRJEB27662	ERR4135366		Korea	Asian	MAFLD
PRJEB27662	ERR4135367		Korea	Asian	MAFLD
PRJEB27662	ERR4135368		Korea	Asian	MAFLD
PRJEB27662	ERR4135369		Korea	Asian	MAFLD
PRJEB27662	ERR4135370		Korea	Asian	MAFLD
PRJEB27662	ERR4135371		Korea	Asian	MAFLD
PRJEB27662	ERR4135372		Korea	Asian	MAFLD
PRJEB27662	ERR4135373		Korea	Asian	MAFLD
PRJEB27662	ERR4135374		Korea	Asian	MAFLD
PRJEB27662	ERR4135375		Korea	Asian	MAFLD
PRJEB27662	ERR4135376		Korea	Asian	MAFLD
PRJEB27662	ERR4135377		Korea	Asian	MAFLD
PRJEB27662	ERR4135378		Korea	Asian	MAFLD
PRJEB27662	ERR4135379		Korea	Asian	MAFLD
PRJEB27662	ERR4135380		Korea	Asian	MAFLD
PRJEB27662	ERR4135381		Korea	Asian	MAFLD
PRJEB27662	ERR4135382		Korea	Asian	MAFLD
PRJEB27662	ERR4135383		Korea	Asian	MAFLD
PRJEB27662	ERR4135384		Korea	Asian	MAFLD
PRJEB27662	ERR4135385		Korea	Asian	MAFLD
PRJEB27662	ERR4135386		Korea	Asian	MAFLD
PRJEB27662	ERR4135387		Korea	Asian	MAFLD
PRJEB27662	ERR4135388		Korea	Asian	MAFLD
PRJEB27662	ERR4135389		Korea	Asian	MAFLD
PRJEB27662	ERR4135390		Korea	Asian	MAFLD
PRJEB27662	ERR4135391		Korea	Asian	MAFLD
PRJEB27662	ERR4135392		Korea	Asian	MAFLD
PRJEB27662	ERR4135393		Korea	Asian	MAFLD
PRJEB27662	ERR4135394		Korea	Asian	MAFLD

PRJEB27662	ERR4135395	Korea	Asian	MAFLD
PRJEB27662	ERR4135396	Korea	Asian	MAFLD
PRJEB27662	ERR4135397	Korea	Asian	MAFLD
PRJEB27662	ERR4135398	Korea	Asian	MAFLD
PRJEB27662	ERR4135399	Korea	Asian	MAFLD
PRJEB27662	ERR4135400	Korea	Asian	MAFLD
PRJEB27662	ERR4135401	Korea	Asian	MAFLD
PRJEB27662	ERR4135402	Korea	Asian	MAFLD
PRJEB27662	ERR4135403	Korea	Asian	MAFLD
PRJEB27662	ERR4135404	Korea	Asian	MAFLD
PRJEB27662	ERR4135405	Korea	Asian	MAFLD
PRJEB27662	ERR4135406	Korea	Asian	MAFLD
PRJEB27662	ERR4135407	Korea	Asian	MAFLD
PRJEB27662	ERR4135408	Korea	Asian	MAFLD
PRJEB27662	ERR4135409	Korea	Asian	MAFLD
PRJEB27662	ERR4135410	Korea	Asian	MAFLD
PRJEB27662	ERR4135411	Korea	Asian	MAFLD
PRJEB27662	ERR4135412	Korea	Asian	MAFLD
PRJEB27662	ERR4135413	Korea	Asian	MAFLD
PRJEB27662	ERR4135414	Korea	Asian	MAFLD
PRJEB27662	ERR4135415	Korea	Asian	MAFLD
PRJEB27662	ERR4135416	Korea	Asian	MAFLD
PRJEB27662	ERR4135417	Korea	Asian	MAFLD
PRJEB27662	ERR4135418	Korea	Asian	MAFLD
PRJEB27662	ERR4135419	Korea	Asian	MAFLD
PRJEB27662	ERR4135420	Korea	Asian	MAFLD
PRJEB27662	ERR4135421	Korea	Asian	MAFLD
PRJEB27662	ERR4135422	Korea	Asian	MAFLD
PRJEB27662	ERR4135423	Korea	Asian	MAFLD
PRJEB27662	ERR4135424	Korea	Asian	MAFLD
PRJEB27662	ERR4135425	Korea	Asian	MAFLD
PRJEB27662	ERR4135426	Korea	Asian	MAFLD
PRJEB27662	ERR4135427	Korea	Asian	MAFLD
PRJEB27662	ERR4135428	Korea	Asian	MAFLD
PRJEB27662	ERR4135429	Korea	Asian	MAFLD
PRJEB27662	ERR4135430	Korea	Asian	MAFLD
PRJEB27662	ERR4135431	Korea	Asian	MAFLD
PRJEB27662	ERR4135432	Korea	Asian	MAFLD
PRJEB40538	ERR4634080	Italy	Caucasian	MAFLD
PRJEB40538	ERR4634087	Italy	Caucasian	MAFLD
PRJEB40538	ERR4634089	Italy	Caucasian	MAFLD
PRJEB40538	ERR4634115	Italy	Caucasian	MAFLD
PRJEB40538	ERR4634117	Italy	Caucasian	MAFLD
PRJEB40538	ERR4634119	Italy	Caucasian	MAFLD
PRJEB40538	ERR4634122	Italy	Caucasian	MAFLD
PRJEB40538	ERR4634124	Italy	Caucasian	MAFLD

PRJEB40538	ERR4634126	Italy	Caucasian	MAFLD
PRJEB40538	ERR4634130	Italy	Caucasian	MAFLD
PRJEB40538	ERR4634131	Italy	Caucasian	MAFLD
PRJEB40538	ERR4634133	Italy	Caucasian	MAFLD
PRJEB40538	ERR4634135	Italy	Caucasian	MAFLD
PRJEB40538	ERR4634146	Italy	Caucasian	MAFLD
PRJEB40538	ERR4634148	Italy	Caucasian	MAFLD
PRJEB40538	ERR4634150	Italy	Caucasian	MAFLD
PRJEB40538	ERR4634151	Italy	Caucasian	MAFLD
PRJEB40538	ERR4634161	Italy	Caucasian	MAFLD
PRJEB40538	ERR4634226	Italy	Caucasian	MAFLD
PRJEB40538	ERR4634228	Italy	Caucasian	MAFLD
PRJEB40538	ERR4634230	Italy	Caucasian	MAFLD
PRJEB40538	ERR4634232	Italy	Caucasian	MAFLD
PRJEB40538	ERR4634234	Italy	Caucasian	MAFLD
PRJEB40538	ERR4634236	Italy	Caucasian	MAFLD
PRJEB40538	ERR4634238	Italy	Caucasian	MAFLD
PRJEB40538	ERR4634240	Italy	Caucasian	MAFLD
PRJEB40538	ERR4634246	Italy	Caucasian	MAFLD
PRJNA540838	SRR10918251	Germany	Caucasian	MAFLD
PRJNA540839	SRR10918252	Germany	Caucasian	MAFLD
PRJNA540840	SRR10918253	Germany	Caucasian	MAFLD
PRJNA540841	SRR10918254	Germany	Caucasian	MAFLD
PRJNA540842	SRR10918255	Germany	Caucasian	MAFLD
PRJNA540843	SRR10918256	Germany	Caucasian	MAFLD
PRJNA540844	SRR10918257	Germany	Caucasian	MAFLD
PRJNA540845	SRR10918258	Germany	Caucasian	MAFLD
PRJNA540846	SRR10918259	Germany	Caucasian	MAFLD
PRJNA540847	SRR10918260	Germany	Caucasian	MAFLD
PRJNA540848	SRR10918261	Germany	Caucasian	MAFLD
PRJNA540849	SRR10918262	Germany	Caucasian	MAFLD
PRJNA540850	SRR10918263	Germany	Caucasian	MAFLD
PRJNA540851	SRR10918264	Germany	Caucasian	MAFLD
PRJNA540852	SRR10918265	Germany	Caucasian	MAFLD
PRJNA540853	SRR10918266	Germany	Caucasian	MAFLD
PRJNA540854	SRR10918267	Germany	Caucasian	MAFLD
PRJNA540855	SRR10918268	Germany	Caucasian	MAFLD
PRJNA540856	SRR10918269	Germany	Caucasian	MAFLD
PRJNA540857	SRR10918270	Germany	Caucasian	MAFLD
PRJNA540858	SRR10918271	Germany	Caucasian	MAFLD
PRJNA540859	SRR10918272	Germany	Caucasian	MAFLD
PRJNA540860	SRR10918273	Germany	Caucasian	MAFLD
PRJNA540861	SRR10918274	Germany	Caucasian	MAFLD
PRJNA540862	SRR10918275	Germany	Caucasian	MAFLD
PRJNA540863	SRR10918276	Germany	Caucasian	MAFLD
PRJNA540864	SRR10918277	Germany	Caucasian	MAFLD

PRJNA540865	SRR10918278			Germany	Caucasian	MAFLD
PRJNA540866	SRR10918279			Germany	Caucasian	MAFLD
PRJNA540867	SRR10918280			Germany	Caucasian	MAFLD
PRJNA540868	SRR10918281			Germany	Caucasian	MAFLD
PRJNA540869	SRR10918282			Germany	Caucasian	MAFLD
PRJNA540870	SRR10918283			Germany	Caucasian	MAFLD
PRJNA540871	SRR10918284			Germany	Caucasian	MAFLD
PRJNA540872	SRR10918285			Germany	Caucasian	MAFLD
PRJNA540873	SRR10918286			Germany	Caucasian	MAFLD
PRJNA540874	SRR10918287			Germany	Caucasian	MAFLD
PRJNA540875	SRR10918288			Germany	Caucasian	MAFLD
PRJNA540876	SRR10918289			Germany	Caucasian	MAFLD
PRJNA540877	SRR10918290			Germany	Caucasian	MAFLD
PRJNA540878	SRR10918291			Germany	Caucasian	MAFLD
PRJNA540879	SRR10918292			Germany	Caucasian	MAFLD
PRJNA540880	SRR10918293			Germany	Caucasian	MAFLD
PRJNA540881	SRR10918294			Germany	Caucasian	MAFLD
PRJNA540752	SRR11193324			Germany	Caucasian	MAFLD
PRJNA540751	SRR11193325			Germany	Caucasian	MAFLD
PRJNA540750	SRR11193326			Germany	Caucasian	MAFLD
PRJNA540749	SRR11193327			Germany	Caucasian	MAFLD
PRJNA540748	SRR11193336			Germany	Caucasian	Health
PRJNA540747	SRR11193337			Germany	Caucasian	Health
PRJNA540746	SRR11193356			Germany	Caucasian	Health
PRJNA540761	SRR11193358			Germany	Caucasian	MAFLD
PRJNA540760	SRR11193362			Germany	Caucasian	MAFLD
PRJNA540745	SRR11193363			Germany	Caucasian	Health
PRJNA540744	SRR11193364			Germany	Caucasian	Health
PRJNA540743	SRR11193365			Germany	Caucasian	Health
PRJNA540742	SRR11193366			Germany	Caucasian	Health
PRJNA540740	SRR11193368			Germany	Caucasian	Health
PRJNA540739	SRR11193369			Germany	Caucasian	Health
PRJNA540741	SRR11193370			Germany	Caucasian	Health
PRJNA540738	SRR11193372			Germany	Caucasian	Health
PRJNA540759	SRR11193373			Germany	Caucasian	MAFLD
PRJNA540758	SRR11193374			Germany	Caucasian	MAFLD
PRJNA540757	SRR11193375			Germany	Caucasian	MAFLD
PRJNA540756	SRR11193376			Germany	Caucasian	MAFLD
PRJNA540755	SRR11193377			Germany	Caucasian	MAFLD
PRJNA540754	SRR11193378			Germany	Caucasian	MAFLD
PRJNA540753	SRR11193379			Germany	Caucasian	MAFLD
PRJNA246121	SRR1273255	58	Female	China	Asian	MAFLD
PRJNA246121	SRR1273259	40	Male	China	Asian	MAFLD
PRJNA246121	SRR1273261	22	Male	China	Asian	MAFLD
PRJNA246121	SRR1273263	37	Male	China	Asian	MAFLD
PRJNA246121	SRR1273265	22	Male	China	Asian	MAFLD

PRJNA246121	SRR1273267	58	Female	China	Asian	MAFLD
PRJNA246121	SRR1273268	56	Female	China	Asian	MAFLD
PRJNA246121	SRR1273270	34	Male	China	Asian	MAFLD
PRJNA246121	SRR1273272	31	Male	China	Asian	MAFLD
PRJNA246121	SRR1273273	49	Female	China	Asian	MAFLD
PRJNA246121	SRR1273275	50	Male	China	Asian	MAFLD
PRJNA246121	SRR1273277	34	Male	China	Asian	MAFLD
PRJNA246121	SRR1273279	41	Male	China	Asian	MAFLD
PRJNA246121	SRR1273281	55	Female	China	Asian	MAFLD
PRJNA246121	SRR1273282	49	Male	China	Asian	MAFLD
PRJNA246121	SRR1273284	50	Female	China	Asian	MAFLD
PRJNA246121	SRR1273285	62	Male	China	Asian	MAFLD
PRJNA246121	SRR1273287	57	Male	China	Asian	MAFLD
PRJNA246121	SRR1273289	37	Male	China	Asian	MAFLD
PRJNA246121	SRR1273290	50	Male	China	Asian	MAFLD
PRJNA246121	SRR1273291	36	Male	China	Asian	MAFLD
PRJNA246121	SRR1273292	53	Male	China	Asian	MAFLD
PRJNA246121	SRR1273294	59	Female	China	Asian	MAFLD
PRJNA246121	SRR1273302	72	Male	China	Asian	MAFLD
PRJNA246121	SRR1273317	37	Female	China	Asian	MAFLD
PRJNA246121	SRR1273337	62	Female	China	Asian	MAFLD
PRJNA246121	SRR1273342	59	Male	China	Asian	MAFLD
PRJNA246121	SRR1273343	59	Male	China	Asian	MAFLD
PRJNA246121	SRR1273344	28	Female	China	Asian	MAFLD
PRJNA246121	SRR1273345	58	Female	China	Asian	MAFLD
PRJNA246121	SRR1273346	71	Male	China	Asian	MAFLD
PRJNA246121	SRR1273347	54	Female	China	Asian	MAFLD
PRJNA246121	SRR1273348	56	Male	China	Asian	MAFLD
PRJNA246121	SRR1273349	46	Female	China	Asian	MAFLD
PRJNA246121	SRR1273350	52	Female	China	Asian	MAFLD
PRJNA246121	SRR1273351	50	Male	China	Asian	MAFLD
PRJNA246121	SRR1273352	72	Female	China	Asian	MAFLD
PRJNA246121	SRR1273353	45	Female	China	Asian	MAFLD
PRJNA246121	SRR1273354	46	Female	China	Asian	MAFLD
PRJNA246121	SRR1273355	50	Female	China	Asian	MAFLD
PRJNA246121	SRR1273356	41	Male	China	Asian	MAFLD
PRJNA246121	SRR1273357	52	Female	China	Asian	MAFLD
PRJNA246121	SRR1273358	56	Female	China	Asian	MAFLD
PRJNA246121	SRR1273359	49	Female	China	Asian	MAFLD
PRJNA246121	SRR1273360	41	Male	China	Asian	MAFLD
PRJNA246121	SRR1273361	48	Male	China	Asian	MAFLD
PRJNA246121	SRR1273362	44	Male	China	Asian	MAFLD
PRJNA246121	SRR1273363	54	Male	China	Asian	MAFLD
PRJNA246121	SRR1273364	38	Female	China	Asian	MAFLD
PRJNA246121	SRR1273365	39	Female	China	Asian	MAFLD
PRJNA246121	SRR1273366	35	Female	China	Asian	MAFLD

PRJNA246121	SRR1273367	38	Female		China	Asian	MAFLD
PRJNA246121	SRR1273368	40	Female		China	Asian	MAFLD
PRJNA246121	SRR1273369	45	Female		China	Asian	Health
PRJNA246121	SRR1273370	39	Female		China	Asian	Health
PRJNA246121	SRR1273371	34	Female		China	Asian	Health
PRJNA246121	SRR1273372	40	Female		China	Asian	Health
PRJNA246121	SRR1273373	38	Female		China	Asian	Health
PRJNA246121	SRR1273374	51	Female		China	Asian	Health
PRJNA246121	SRR1273375	42	Female		China	Asian	Health
PRJNA246121	SRR1273376	43	Female		China	Asian	Health
PRJNA246121	SRR1273377	50	Female		China	Asian	Health
PRJNA246121	SRR1273378	41	Male		China	Asian	Health
PRJNA246121	SRR1273379	52	Female		China	Asian	Health
PRJNA246121	SRR1273380	44	Female		China	Asian	Health
PRJNA246121	SRR1273381	41	Female		China	Asian	Health
PRJNA246121	SRR1273382	40	Male		China	Asian	Health
PRJNA246121	SRR1273383	42	Female		China	Asian	Health
PRJNA246121	SRR1273384	38	Male		China	Asian	Health
PRJNA246121	SRR1273385	40	Female		China	Asian	Health
PRJNA246121	SRR1273389	26	Female		China	Asian	Health
PRJNA246121	SRR1273390	41	Female		China	Asian	Health
PRJNA246121	SRR1273391	40	Female		China	Asian	Health
PRJNA246121	SRR1273392	50	Female		China	Asian	Health
PRJNA246121	SRR1273393	39	Female		China	Asian	Health
PRJNA246121	SRR1273394	43	Female		China	Asian	Health
PRJNA246121	SRR1273396	33	Female		China	Asian	Health
PRJNA246121	SRR1273397	35	Female		China	Asian	Health
PRJNA246121	SRR1273398	40	Male		China	Asian	Health
PRJNA246121	SRR1273399	43	Female		China	Asian	Health
PRJNA246121	SRR1273400	35	Female		China	Asian	Health
PRJNA246121	SRR1273401	42	Female		China	Asian	Health
PRJNA246121	SRR1273402	42	Female		China	Asian	Health
PRJNA246121	SRR1273403	28	Male		China	Asian	Health
PRJNA246121	SRR1273404	47	Female		China	Asian	Health
PRJNA401981	SRR6024843	41	Female	22.2	Italy	Caucasian	Health
PRJNA401981	SRR6024844	44	Female	21.4	Italy	Caucasian	Health
PRJNA401981	SRR6024845	49	Female	19.1	Italy	Caucasian	Health
PRJNA401981	SRR6024846	46	Female	24.6	Italy	Caucasian	Health
PRJNA401981	SRR6024847	55	Female	24.4	Italy	Caucasian	Health
PRJNA401981	SRR6024848	51	Female	23.1	Italy	Caucasian	Health
PRJNA401981	SRR6024849	62	Female	22.1	Italy	Caucasian	Health
PRJNA401981	SRR6024850	44	Female	23.1	Italy	Caucasian	Health
PRJNA401981	SRR6024853	35	Male	24.7	Italy	Caucasian	Health
PRJNA401981	SRR6024854	38	Male	22.2	Italy	Caucasian	Health
PRJNA401981	SRR6024855	42	Male	24.2	Italy	Caucasian	Health
PRJNA401981	SRR6024856	38	Male	22.1	Italy	Caucasian	Health

PRJNA401981	SRR6024857	58	Male	22.6	Italy	Caucasian	Health
PRJNA401981	SRR6024858	51	Male	20.5	Italy	Caucasian	Health
PRJNA401981	SRR6024859	56	Male	25.8	Italy	Caucasian	Health
PRJNA401981	SRR6024860	60	Male	19.4	Italy	Caucasian	Health
PRJNA401981	SRR6024861	46	Male	24.8	Italy	Caucasian	Health
PRJNA401981	SRR6024862	63	Male	23.6	Italy	Caucasian	Health
PRJNA414683	SRR6185168	59	Male	28.4	New Zealand	Caucasian	Health
PRJNA414683	SRR6185180	40	Male	27.1	New Zealand	Caucasian	Health
PRJNA414683	SRR6185189	59	Male	28.4	New Zealand	Caucasian	Health
PRJNA414683	SRR6185204	59	Male	28.4	New Zealand	Caucasian	Health
PRJNA414683	SRR6185211	40	Male	27.1	New Zealand	Caucasian	Health
PRJNA414683	SRR6185233	33	Female	26.5	New Zealand	Caucasian	Health
PRJNA414683	SRR6185239	34	Male	27.3	New Zealand	Caucasian	Health
PRJNA414683	SRR6185241	59	Male	28.4	New Zealand	Caucasian	Health
PRJNA414683	SRR6185244	33	Female	26.5	New Zealand	Caucasian	Health
PRJNA414683	SRR6185260	40	Male	27.1	New Zealand	Caucasian	Health
PRJNA414683	SRR6185268	33	Female	26.5	New Zealand	Caucasian	Health
PRJNA414683	SRR6185271	34	Male	27.3	New Zealand	Caucasian	Health
PRJNA414683	SRR6185276	40	Male	27.1	New Zealand	Caucasian	Health
PRJNA414683	SRR6185280	34	Male	27.3	New Zealand	Caucasian	Health
PRJNA414683	SRR6185287	33	Female	26.5	New Zealand	Caucasian	Health
PRJNA414683	SRR6185294	34	Male	27.3	New Zealand	Caucasian	Health
PRJNA518731	SRR8518981				New Zealand	Caucasian	MAFLD
PRJNA518731	SRR8518983				New Zealand	Caucasian	MAFLD
PRJNA518731	SRR8518987				New Zealand	Caucasian	MAFLD
PRJNA518731	SRR8518988				New Zealand	Caucasian	MAFLD
PRJNA518731	SRR8518990				New Zealand	Caucasian	MAFLD
PRJNA518731	SRR8518998				New Zealand	Caucasian	MAFLD
PRJNA518731	SRR8519001				New Zealand	Caucasian	MAFLD
PRJNA518731	SRR8519002				New Zealand	Caucasian	MAFLD
PRJNA518731	SRR8519003				New Zealand	Caucasian	MAFLD
PRJNA518731	SRR8519013				New Zealand	Caucasian	MAFLD
PRJNA518731	SRR8519019				New Zealand	Caucasian	MAFLD
PRJNA518731	SRR8519022				New Zealand	Caucasian	MAFLD
PRJNA518731	SRR8519023				New Zealand	Caucasian	MAFLD
PRJNA518731	SRR8519028				New Zealand	Caucasian	MAFLD
PRJNA518731	SRR8519029				New Zealand	Caucasian	MAFLD
PRJNA518731	SRR8519031				New Zealand	Caucasian	MAFLD
PRJNA518731	SRR8519032				New Zealand	Caucasian	MAFLD
PRJNA518731	SRR8519036				New Zealand	Caucasian	MAFLD
PRJNA518731	SRR8519038				New Zealand	Caucasian	MAFLD
PRJNA518731	SRR8519041				New Zealand	Caucasian	MAFLD
PRJNA518731	SRR8519046				New Zealand	Caucasian	MAFLD
PRJNA518731	SRR8519050				New Zealand	Caucasian	MAFLD
PRJNA518731	SRR8519051				New Zealand	Caucasian	MAFLD
PRJNA518731	SRR8519056				New Zealand	Caucasian	MAFLD

PRJNA518731	SRR8519057	New Zealand	Caucasian	MAFLD
PRJNA518731	SRR8519062	New Zealand	Caucasian	MAFLD
PRJNA518731	SRR8519066	New Zealand	Caucasian	MAFLD
PRJNA518731	SRR8519072	New Zealand	Caucasian	MAFLD
PRJNA518731	SRR8519076	New Zealand	Caucasian	MAFLD
PRJNA518731	SRR8519086	New Zealand	Caucasian	MAFLD
PRJNA518731	SRR8519089	New Zealand	Caucasian	MAFLD
PRJNA518731	SRR8519093	New Zealand	Caucasian	MAFLD
PRJNA518731	SRR8519099	New Zealand	Caucasian	MAFLD
PRJNA518731	SRR8519101	New Zealand	Caucasian	MAFLD
PRJNA518731	SRR8519103	New Zealand	Caucasian	MAFLD
PRJNA518731	SRR8519104	New Zealand	Caucasian	MAFLD
PRJNA540790	SRR9001634	Germany	Caucasian	MAFLD
PRJNA540774	SRR9001635	Germany	Caucasian	MAFLD
PRJNA540769	SRR9001639	Germany	Caucasian	Health
PRJNA540768	SRR9001640	Germany	Caucasian	Health
PRJNA540767	SRR9001641	Germany	Caucasian	Health
PRJNA540792	SRR9001642	Germany	Caucasian	MAFLD
PRJNA540791	SRR9001643	Germany	Caucasian	MAFLD
PRJNA540770	SRR9001644	Germany	Caucasian	MAFLD
PRJNA540772	SRR9001646	Germany	Caucasian	MAFLD
PRJNA540775	SRR9001651	Germany	Caucasian	MAFLD
PRJNA540773	SRR9001652	Germany	Caucasian	MAFLD
PRJNA540793	SRR9001653	Germany	Caucasian	MAFLD
PRJNA540794	SRR9001654	Germany	Caucasian	MAFLD
PRJNA540795	SRR9001655	Germany	Caucasian	MAFLD
PRJNA540796	SRR9001656	Germany	Caucasian	MAFLD
PRJNA540797	SRR9001657	Germany	Caucasian	MAFLD
PRJNA540798	SRR9001658	Germany	Caucasian	MAFLD
PRJNA540799	SRR9001659	Germany	Caucasian	MAFLD
PRJNA540800	SRR9001660	Germany	Caucasian	MAFLD
PRJNA540810	SRR9001661	Germany	Caucasian	MAFLD
PRJNA540811	SRR9001662	Germany	Caucasian	MAFLD
PRJNA540782	SRR9001663	Germany	Caucasian	MAFLD
PRJNA540783	SRR9001664	Germany	Caucasian	MAFLD
PRJNA540780	SRR9001665	Germany	Caucasian	MAFLD
PRJNA540781	SRR9001666	Germany	Caucasian	MAFLD
PRJNA540778	SRR9001667	Germany	Caucasian	MAFLD
PRJNA540779	SRR9001668	Germany	Caucasian	MAFLD
PRJNA540776	SRR9001669	Germany	Caucasian	MAFLD
PRJNA540777	SRR9001670	Germany	Caucasian	MAFLD
PRJNA540784	SRR9001671	Germany	Caucasian	MAFLD
PRJNA540785	SRR9001672	Germany	Caucasian	MAFLD
PRJNA540771	SRR9001674	Germany	Caucasian	MAFLD
PRJNA540762	SRR9001676	Germany	Caucasian	Health
PRJNA540789	SRR9001684	Germany	Caucasian	MAFLD

PRJNA540788	SRR9001685	Germany	Caucasian	MAFLD
PRJNA540787	SRR9001686	Germany	Caucasian	MAFLD
PRJNA540786	SRR9001687	Germany	Caucasian	MAFLD
PRJNA540804	SRR9001688	Germany	Caucasian	MAFLD
PRJNA540803	SRR9001689	Germany	Caucasian	MAFLD
PRJNA540801	SRR9001690	Germany	Caucasian	MAFLD
PRJNA540806	SRR9001691	Germany	Caucasian	MAFLD
PRJNA540805	SRR9001692	Germany	Caucasian	MAFLD
PRJNA540823	SRR9001696	Germany	Caucasian	MAFLD
PRJNA540824	SRR9001697	Germany	Caucasian	MAFLD
PRJNA540819	SRR9001698	Germany	Caucasian	MAFLD
PRJNA540820	SRR9001699	Germany	Caucasian	MAFLD
PRJNA540821	SRR9001700	Germany	Caucasian	MAFLD
PRJNA540822	SRR9001701	Germany	Caucasian	MAFLD
PRJNA540807	SRR9001702	Germany	Caucasian	MAFLD
PRJNA540808	SRR9001703	Germany	Caucasian	MAFLD
PRJNA540809	SRR9001704	Germany	Caucasian	MAFLD
PRJNA540818	SRR9001705	Germany	Caucasian	MAFLD
PRJNA540837	SRR9001710	Germany	Caucasian	MAFLD
PRJNA540802	SRR9001711	Germany	Caucasian	MAFLD
PRJNA540816	SRR9001712	Germany	Caucasian	MAFLD
PRJNA540815	SRR9001713	Germany	Caucasian	MAFLD
PRJNA540814	SRR9001714	Germany	Caucasian	MAFLD
PRJNA540813	SRR9001715	Germany	Caucasian	MAFLD
PRJNA540812	SRR9001716	Germany	Caucasian	MAFLD
PRJNA540835	SRR9001717	Germany	Caucasian	MAFLD
PRJNA540834	SRR9001718	Germany	Caucasian	MAFLD
PRJNA540827	SRR9001719	Germany	Caucasian	MAFLD
PRJNA540826	SRR9001720	Germany	Caucasian	MAFLD
PRJNA540829	SRR9001721	Germany	Caucasian	MAFLD
PRJNA540828	SRR9001722	Germany	Caucasian	MAFLD
PRJNA540831	SRR9001723	Germany	Caucasian	MAFLD
PRJNA540830	SRR9001724	Germany	Caucasian	MAFLD
PRJNA540833	SRR9001725	Germany	Caucasian	MAFLD
PRJNA540832	SRR9001726	Germany	Caucasian	MAFLD
PRJNA540765	SRR9001727	Germany	Caucasian	Health
PRJNA540766	SRR9001728	Germany	Caucasian	Health
PRJNA540836	SRR9001731	Germany	Caucasian	MAFLD
PRJNA540763	SRR9001733	Germany	Caucasian	Health
PRJNA540764	SRR9001734	Germany	Caucasian	Health
PRJNA540825	SRR9016647	Germany	Caucasian	MAFLD
PRJNA540817	SRR9016650	Germany	Caucasian	MAFLD

Supplementary table S3. Comparison of network topological data for gut microbiota between Healthy and MAFLD individuals

	Health	MAFLD
**No. Node**	131	130
**No. Edge**	432	404
**Average degree**	6.6	5.82
**Average path length**	0.79	1.17
**Negative Edge Ratio**	0.58	0.55
**Robustness (50%)**	0.47	0.42

Supplementary Table S4. Differential Network Parameters of P.copri Among Ethnicities Based on ET-P.

	Asians	Caucaians
**Size**	36.3596	31.8225
**Degree**	57	66
**Weighted Degree**	14.1286	6.5946
**Closeness Centrality**	0.565217	0.577103
**Betweenness Centrality**	0.003649	0.00666
**PageRank**	0.015287	0.006528
**Clustering Coefficient**	0.408521	0.255944
**Eigenvector Centrality**	0.364104	0.34376
